# Identification of Dmrt2a downstream genes during zebrafish early development using a timely controlled approach

**DOI:** 10.1186/s12861-018-0173-5

**Published:** 2018-06-19

**Authors:** Rita Alexandra Pinto, José Almeida-Santos, Raquel Lourenço, Leonor Saúde

**Affiliations:** 1 Instituto de Medicina Molecular, Faculdade de Medicina, Universidade de Lisboa, Avenida Professor Egas Moniz, 1649-028 Lisboa, Portugal; 2 Instituto de Medicina Molecular e Instituto de Histologia e Biologia do Desenvolvimento, Faculdade de Medicina, Universidade de Lisboa, Avenida Professor Egas Moniz, 1649-028 Lisboa, Portugal; 3 Present address: Instituto Gulbenkian de Ciência, 2780-156 Oeiras, Portugal; 4 Present address: CEDOC, NOVA Medical School, Universidade Nova de Lisboa, 1150-190 Lisboa, Portugal

**Keywords:** Dmrt2a, *dmrt2a* transgenic, Dmrt2b, TALEN mutants, Microarrays, Zebrafish

## Abstract

**Background:**

Dmrt2a is a zinc finger like transcription factor with several roles during zebrafish early development: left-right asymmetry, synchronisation of the somite clock genes and fast muscle differentiation. Despite the described functions, Dmrt2a mechanism of action is unknown. Therefore, with this work, we propose to identify Dmrt2a downstream genes during zebrafish early development.

**Results:**

We generated and validated a heat-shock inducible transgenic line, to timely control *dmrt2a* overexpression, and *dmrt2a* mutant lines.

We characterised *dmrt2a* overexpression phenotype and verified that it was very similar to the one described after knockdown of this gene, with left-right asymmetry defects and desynchronisation of somite clock genes. Additionally, we identified a new phenotype of somite border malformation.

We generated several *dmrt2a* mutant lines, but we only detected a weak to negligible phenotype. As *dmrt2a* has a paralog gene, *dmrt2b*, with similar functions and expression pattern, we evaluated the possibility of redundancy. We found that *dmrt2b* does not seem to compensate the lack of *dmrt2a*. Furthermore, we took advantage of one of our mutant lines to confirm *dmrt2a* morpholino specificity, which was previously shown to be a robust knockdown tool in two independent studies.

Using the described genetic tools to perform and validate a microarray, we were able to identify six genes downstream of Dmrt2a: *foxj1b*, *pxdc1b, cxcl12b, etv2*, *foxc1b* and *cyp1a*.

**Conclusions:**

In this work, we generated and validated several genetic tools for *dmrt2a* and identified six genes downstream of this transcription factor. The identified genes will be crucial to the future understanding of Dmrt2a mechanism of action in zebrafish.

**Electronic supplementary material:**

The online version of this article (10.1186/s12861-018-0173-5) contains supplementary material, which is available to authorized users.

## Background

The DMRT (Doublesex and Mab3 Related Transcription factors) family of zinc finger like transcription factors has been classically associated with sexual determination and differentiation. Although these functions remain fairly conserved in the different animals studied so far, during evolution different members of this family acquired different functions, such as nervous system development, somitogenesis and left-right asymmetry establishment [1].

When considering the DMRT family, Dmrt2a is particularly notorious because it was the first DMRT protein to be identified with a role apart from sexual determination and differentiation. Meng et al. were the first to describe *dmrt2a* expression pattern in the somites, in zebrafish and mouse, and to propose its role in somitogenesis [2]. Later, *dmrt2a* was found in the Left-Right Organiser (LRO) of zebrafish and chicken, and was found to be necessary not only for somitogenesis but also to the synchronisation of the somite clock genes, left-right asymmetry and more recently, to fast muscle differentiation [3–6]. Moreover, a fish-specific paralog gene – *dmrt2b* – was also identified with similar roles regarding somitogenesis and left-right asymmetry establishment [7, 8].

In mouse, DMRT2 was only associated with somitogenesis-related processes. It was proposed that this transcription factor could affect extracellular matrix components such as LAMININ-1 and, it was further demonstrated that DMRT2 could act in a PAX3/DMRT2/MYF5 regulatory cascade at the onset of myogenesis [9, 10].

On the other hand in zebrafish, several roles were described for Dmrt2a, but little is known about its targets and signalling pathway. Until now, only two players are known to interact with *dmrt2a*, at the level of its 3’UTR: Celf1 and miR-203a [5, 6]. Celf1 is an RNA-binding protein that promotes *dmrt2a* mRNA decay. Upon overexpression of *celf1*, *dmrt2a* mRNA levels are decreased, and problems in the synchronisation of the somite clock genes and in left-right asymmetry establishment arise [5]. miR-203a is a microRNA, which also regulates *dmrt2a* mRNA levels, being needed to ensure correct fast muscle development [6].

In this work, our main goal was to identify Dmrt2a downstream genes in order to clarify how this transcription factor controls so many different developmental processes in zebrafish. In order to accomplish this goal, we generated a heat-shock inducible transgenic line that allowed us to induce a timely controlled *dmrt2a* overexpression, and using the TALEN technology, we generated *dmrt2a* stable mutant lines. Taking advantage of one of the *dmrt2a* mutant lines, we were able to validate the specificity of the previously used *dmrt2a* morpholino (MO) [3, 5]. Using our tools, we performed and validated a microarray experiment. We identified *foxj1b*, *pxdc1b, cxcl12b, etv2*, *foxc1b* and *cyp1a* as genes downstream of Dmrt2a. Our findings give the first steps in order to understand Dmrt2a mechanism of action during zebrafish early development.

## Results

### Generation of a heat-shock inducible transgenic line to timely overexpress *dmrt2a*

**Fig. 1:**
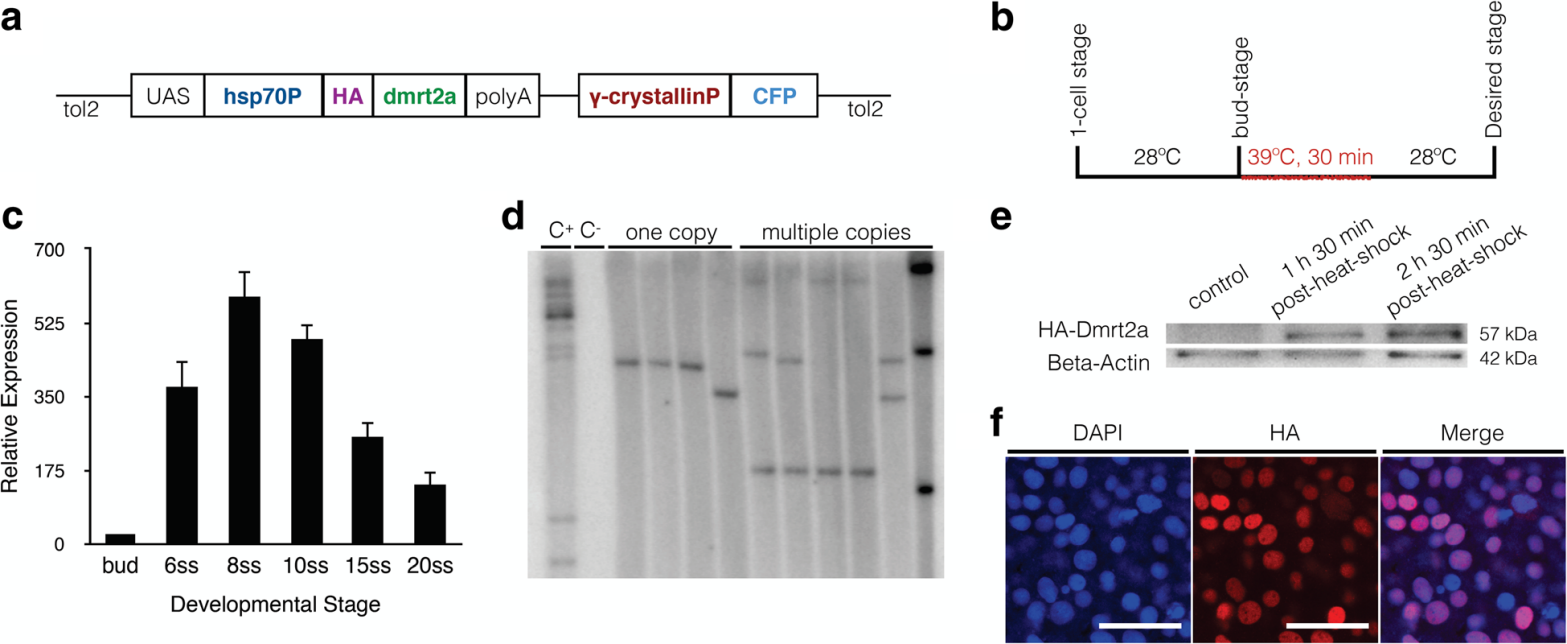
Generation and characterisation of the Tg(hsp70:HA-*dmrt2a*) line. **a** Transgenic construct design. **b** Heat-shock protocol: embryos were raised at 28 °C and the heat-shock performed for 30 min at 39 °C. **c** qPCR analysis showing that *dmrt2a* transcripts are present from bud-stage until 20-somite stage. **d** Southern blot hybridisation using a ^32^P-labelled γ-crystallin probe showing F0 fish with multiple bands (C^+^, positive control), wildtype fish (C^−^, negative control), and F1 generation fish with only one copy of the transgene or multiple copies. **e** The HA-Dmrt2a protein is produced with the correct molecular weight (57 kDa). **f** The fusion protein HA-Dmrt2a (red) co-localises with the nucleus (blue). h: hours, min: minutes, ss: somite stage. Scale bar: 30 μm

We generated a heat-shock inducible transgenic line Tg(hsp70:HA-*dmrt2a*) (Fig. 1a) to overexpress *dmrt2a* in a timely controlled manner. We induced a heat-shock (Fig. 1b) at bud-stage given that this time-point coincides with *dmrt2a* early expression (Fig. 1c), marks the beginning of somitogenesis [11] and precedes the formation of zebrafish LRO [12].

To ensure we had a homogenous transgenic population, we selected the fish with only one copy of the transgene (Fig. 1d). We confirmed that after a heat-shock, the HA-Dmrt2a fusion protein was produced with the correct molecular weight (Fig. 1e) and translocated into the nucleus (Fig. 1f). This translocation was controlled by the endogenous nuclear localisation signal contained within Dmrt2a DM domain [13].

### Transient increase of *dmrt2a* expression produces a similar phenotype to *dmrt2a* loss-of-function

We used the Tg(hsp70:HA-*dmrt2a*) to investigate the impact of transiently overexpressing *dmrt2a* during the developmental processes known to be regulated by this gene: left-right asymmetry and somite formation [3, 5].

**Fig. 2:**
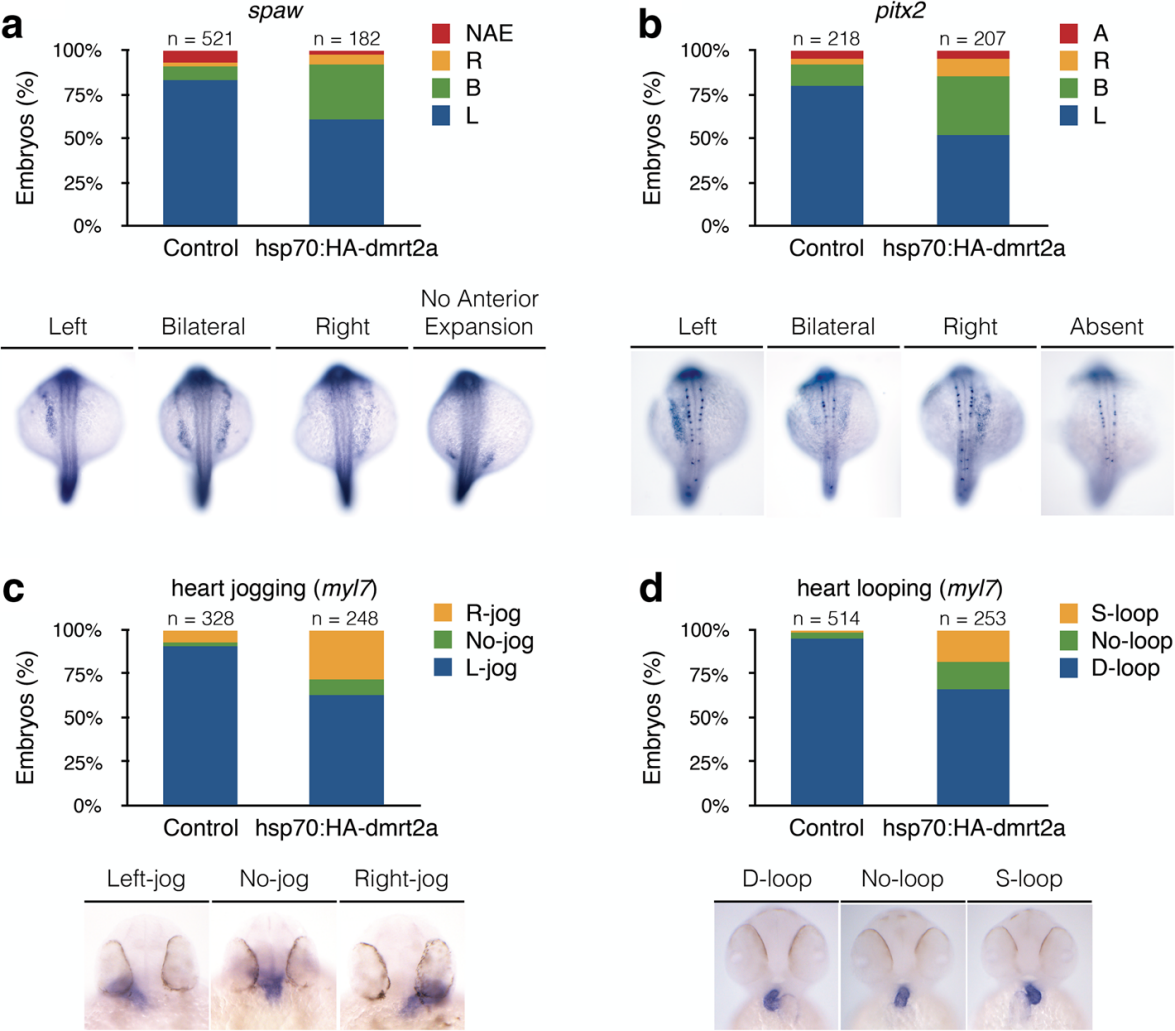
Characterisation of *dmrt2a* overexpression phenotype: left-right asymmetry. **a** Left-sided gene *spaw* (20-somite stage). **b** Left-sided gene *pitx2* (20-somite stage). **c** Heart jogging at 30 hpf, using a *myl7* probe. **d** Heart looping at 48 hpf, using a *myl7* probe. The embryos shown illustrate the different phenotypes obtained. L: left, R: right, B: bilateral, NAE: no anterior expansion, A: absent, hpf: hours post-fertilisation

In the context of left-right asymmetry establishment, *dmrt2a* overexpression led to an increased bilateral expression of the laterality markers, *spaw* and *pitx2*, when compared to controls (Fig. 2a, b). As Nodal signalling promotes the left-jog of the heart tube [14] and given that *spaw* and *pitx2* became bilateral upon *dmrt2a* overexpression, we expected an equivalent percentage of no-jog heart phenotype. However, we obtained an increased percentage of right-jog phenotype, when compared to controls (Fig. 2c). Although bilaterally expressed, small differences in *spaw* levels, between the left and right sides of the lateral plate mesoderm, could be sufficient to drive an asymmetric heart jogging. During heart tube morphogenesis, jogging to the left side is followed by looping to the right side of the embryo (i.e., D-loop). When compared to controls, we obtained a higher percentage of no-loop and S-loop (i.e., left-sided loop) heart phenotypes after *dmrt2a* overexpression, showing a lack of agreement between jogging and looping (Fig. 2d). These results further support the idea that heart looping can be a Nodal independent mechanism [15].

**Fig. 3:**
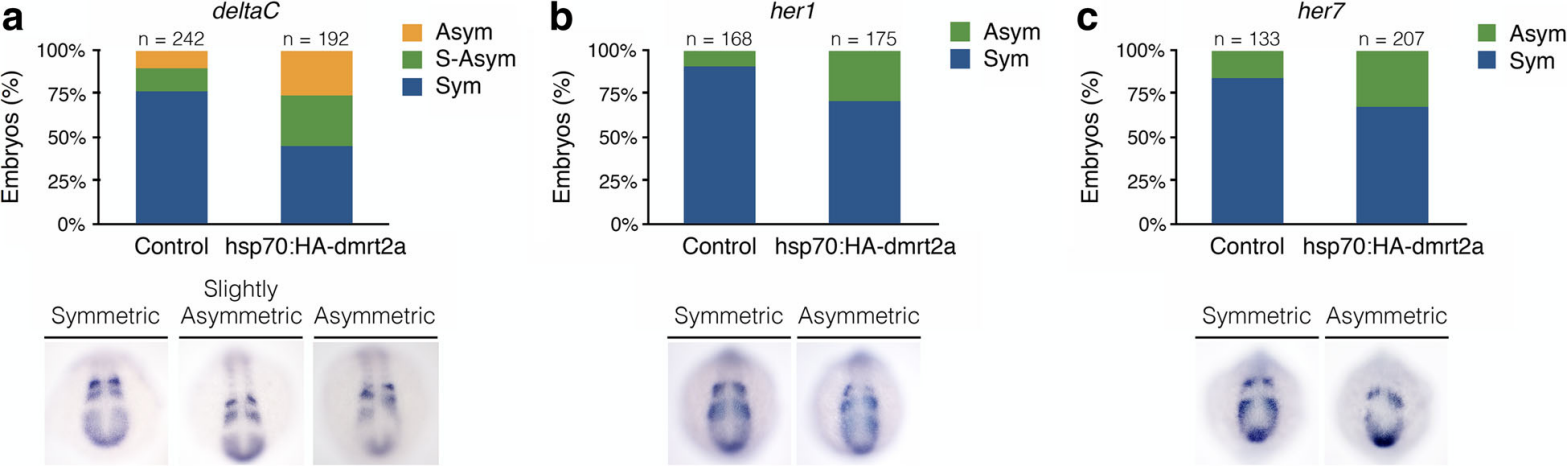
Characterisation of *dmrt2a* overexpression phenotype: somite clock genes. **a** *deltaC* (8-somite stage). **b** *her1* (8-somite stage). **c** *her7* (8-somite stage). The embryos shown illustrate the different phenotypes obtained. Sym: symmetric, S-Asym: slightly asymmetric, Asym: asymmetric

**Fig. 4:**
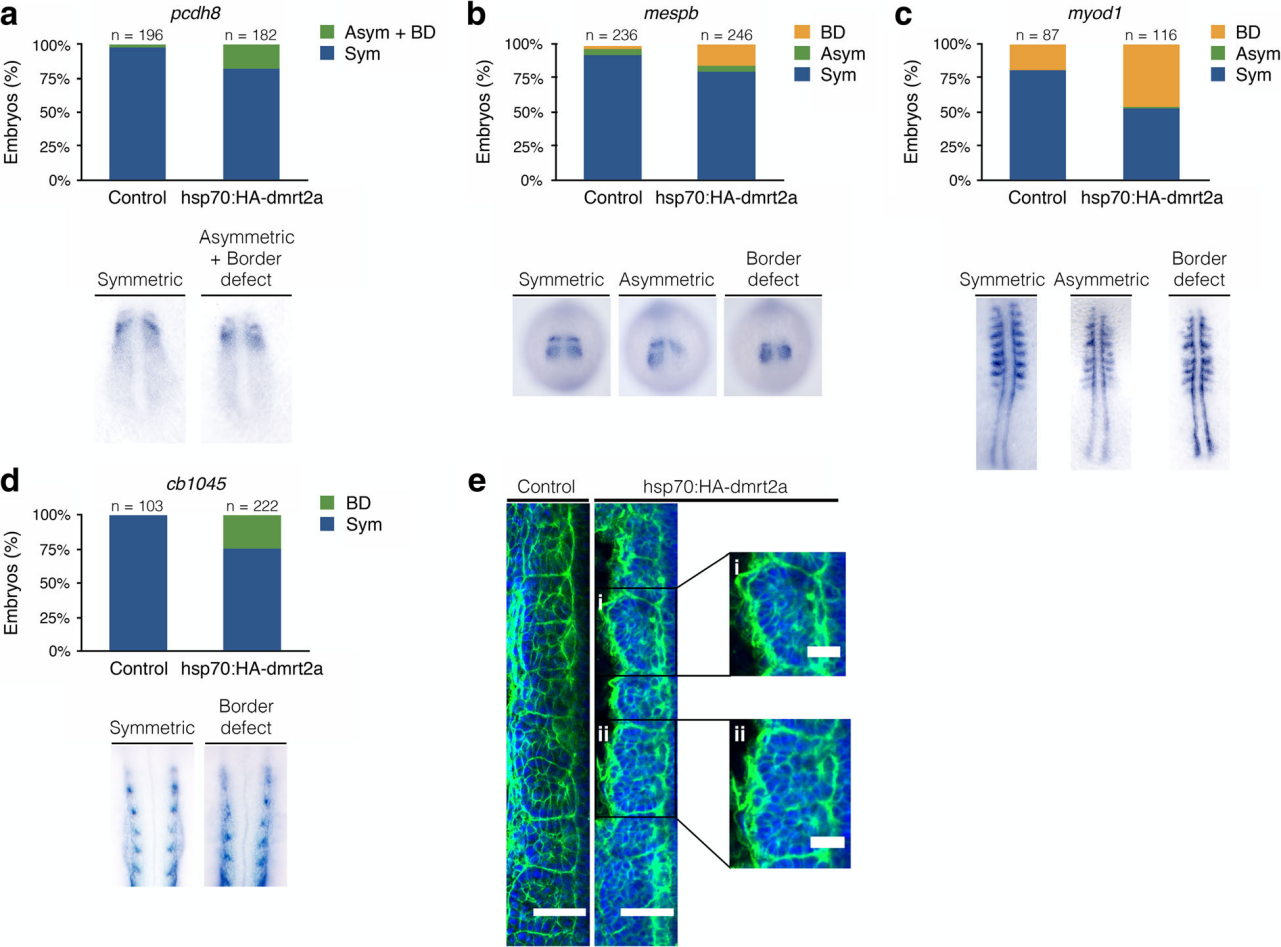
Characterisation of *dmrt2a* overexpression phenotype: somite differentiation and formation markers. **a** Somite differentiation gene *pcdh8* (8-somite stage). **b** Somite boundary gene *mespb* (8-somite stage). **c** Muscle differentiation marker *myod1* (14-somite stage). **d** Somite differentiation gene *cb1045* (24 hpf). The embryos shown illustrate the different phenotypes obtained. **e** F-actin staining in control (*n* = 40/42) and in Tg(hsp70:HA-*dmrt2a*) after *dmrt2a* overexpression (*n* = 22/51) where lack of intersomitic borders is observed (**i**, **ii**). Embryos between 8 and 10-somite stage were used. Sym: symmetric, Asym: asymmetric, BD: border defect, hpf: hours post-fertilisation. Scale bar: 45 μm (**e**) and 20 μm (zoomed panels **i** and **ii**)

In the context of bilateral somite formation, we obtained more embryos with a desynchronised expression of the somite clock genes, *deltaC*, *her1* and *her7* after *dmrt2a* overexpression, when compared to controls (Fig. 3a-c). To evaluate the impact of this desynchronisation in somitogenesis, we evaluated *pcdh8*, *mespb*, *myod1* and *cb1045* expression patterns. All these markers revealed defects in somite morphogenesis (Fig. 4a-d) that can be due to poorly defined or absent intersomitic borders, as we observed (Fig. 4e).

Therefore, *dmrt2a* overexpression produces a similar phenotype to the one previously described when this gene is knockdown [3, 5], suggesting a need to fine tune *dmrt2a* levels during early development. Additionally, we observed a robust new phenotype in the formation of somite boundaries.

### A mild to negligible phenotype is observed in *dmrt2a* mutants

**Fig. 5:**
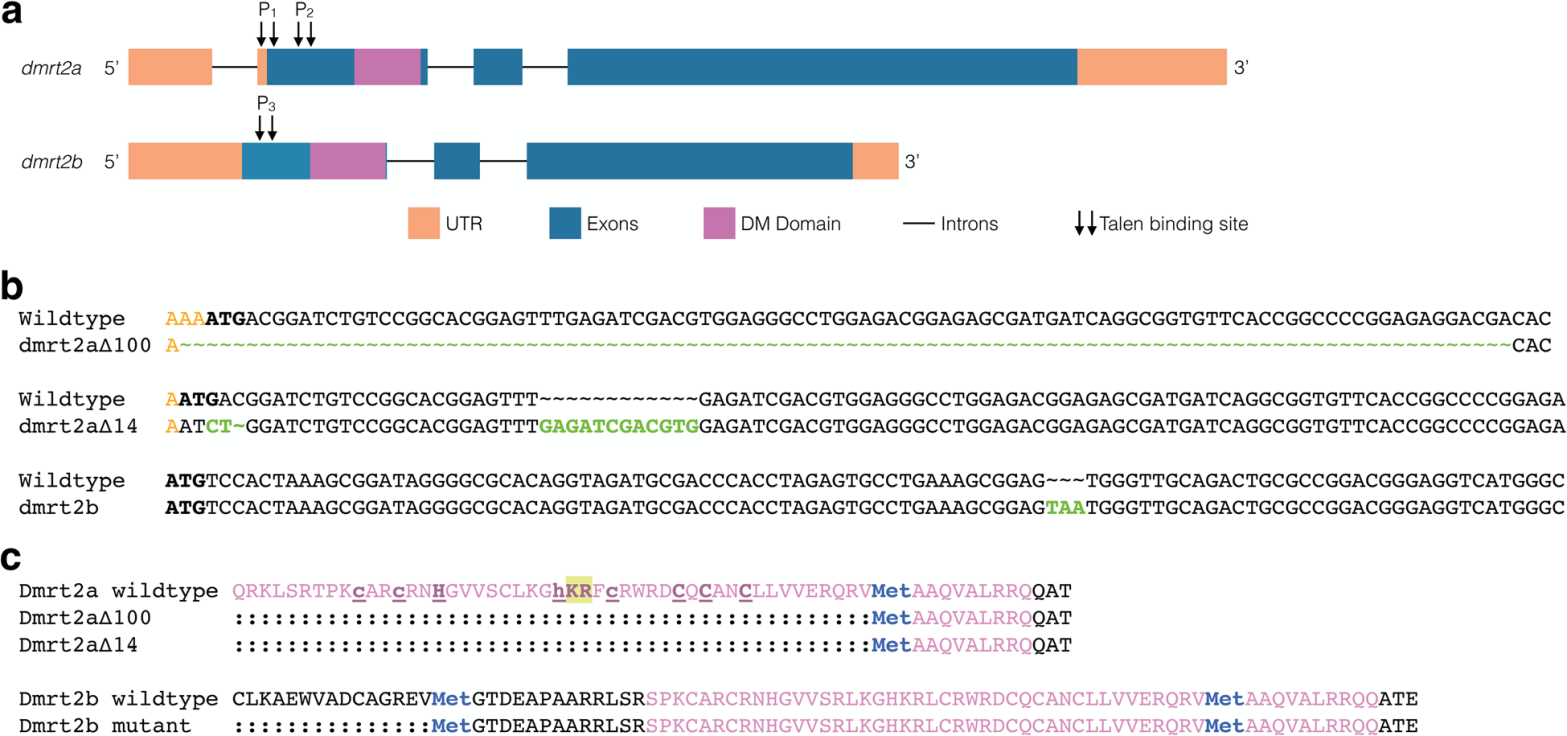
Generation of *dmrt2a* and *dmrt2b* mutant lines. **a** Representation of *dmrt2a* and *dmrt2b* genes. Depiction of the binding site of the three different TALEN pairs (P_1_, P_2_ and P_3_). **b** Representation of the mutations obtained. In bold caps is represented the start codon, in green the indels and in orange the 3’end of the 5’UTR. **c** Alignment between the wildtype sequence of Dmrt2a and Dmrt2b proteins and the expected sequence in the different mutants obtained. In purple is represented the DM domain and in blue the position of putative alternative start codons. In the DM domain of Dmrt2a wildtype protein, the crucial amino acid residues for nuclear translocation and DNA-binding are highlighted, as described [13]: in underlined bold lowercase are represented the ligand-binding residues from the first zinc-binding site and, in underlined bold capital letters the residues from the second zinc-binding site. The two strongly basic residues (K77 and R78) required for nuclear translocation are shaded in yellow. UTR: Untranslated Region, DM: Doublesex and Mab3

**Fig. 6:**
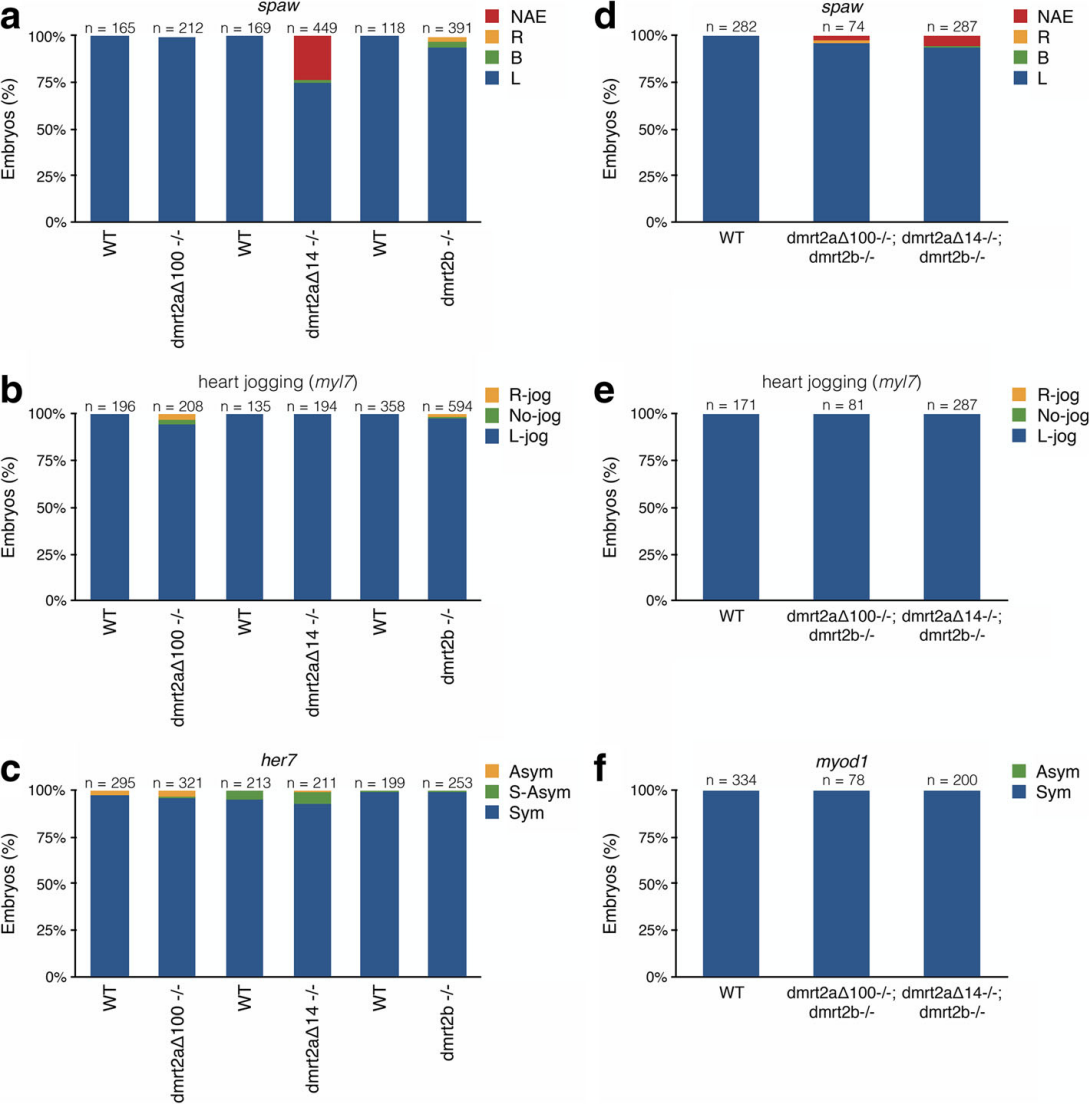
Characterisation of the single and double *dmrt2a* and *dmrt2b* mutants phenotype. **a, b, c** Characterisation of *dmrt2a*^*Δ100−/−*^, *dmrt2a*^*Δ14−/−*^ and *dmrt2b*^*−/−*^ mutant phenotype using: (**a**) left-sided gene *spaw* (20-somite stage), (**b**) *myl7* for heart jogging at 30 hpf and (**c**) *her7*, a cycling gene (8-somite stage). **d, e, f** Characterisation of *dmrt2a*^*Δ100−/−*^;*dmrt2b*^*−/−*^ and *dmrt2a*^*Δ14−/−*^*;dmrt2b*^*−/−*^ double mutants phenotype using: (**d**) left-sided gene *spaw* (20-somite stage), (**e**) *myl7* for heart jogging at 30 hpf and (**f**) *myod1,* a muscle differentiation marker (14-somite stage). L: left, R: right, B: bilateral, NAE: no anterior expansion, Sym: symmetric, S-Asym: slightly asymmetric, Asym: asymmetric, WT: wildtype, hpf: hours post-fertilisation

In order to produce stable *dmrt2a* mutant lines, we used the TALEN technology (Fig. 5a and Additional file 1: Figure S1a, b). We obtained two different mutants: *dmrt2a*^*Δ100*^, which lacks the 3’end of the 5’UTR, the start codon and part of the first exon spanning a 100 bp region; and *dmrt2a*^*Δ14*^, which also lacks the start codon and has a 12 bp duplication in the first exon (Fig. 5b). *dmrt2a* mutants in homozygosity were viable and were used to characterise the loss-of-function phenotype. We observed that *dmrt2a*^*Δ14−/−*^ had a mild phenotype regarding *spaw* expression pattern and *dmrt2a*^*Δ100−/−*^ only had a very mild phenotype regarding heart jogging (Fig. 6a, b). In both cases, *her7* expression pattern was unaffected (Fig. 6c).

As *dmrt2a* has a paralog gene – *dmrt2b* – with a similar expression pattern in somites and with described roles in somitogenesis and left-right asymmetry establishment [7, 8], we wanted to verify if, in the absence of *dmrt2a*, *dmrt2b* could compensate its lack. Therefore, using the TALEN technology, we also produced a *dmrt2b* mutant, which has an early stop codon (p.E22_W23insX, Fig. 5a, b and Additional file 1: Figure S1c). Also in homozygosity, *dmrt2b* mutants were viable and presented an even milder phenotype than *dmrt2a* mutants in the different evaluated markers (Fig. 6a-c). We proceeded with the generation of double mutants: *dmrt2a*^*Δ100*^*;dmrt2b* and *dmrt2a*^*Δ14*^*;dmrt2b*. When analysing the double mutants, we observed that: (1) the *spaw* phenotype in *dmrt2a*^*Δ14−/−*^*;dmrt2b*^*−/−*^ was milder than in the single *dmrt2a*^*Δ14−/−*^ mutant, and (2) the heart jogging phenotype present in *dmrt2a*^*Δ100−/−*^ single mutant, disappeared in the double mutant context (Fig. 6d, e). In both cases, *myod1* expression pattern was unaffected (Fig. 6f). As the left-right asymmetry phenotype observed was not consistent between single and double mutants and, as previously reported, zebrafish wildtype embryos can have up to 10% left-right asymmetry defects [16], we considered our phenotypes negligible.

The weak or negligible phenotype observed in our mutant lines can have several explanations, such as the presence of alternative ATGs, the production of Dmrt2 proteins with residual function or the activation of compensatory genes.

Regarding the first hypothesis, we evaluated all the possible alternative ATGs present from the 5’end of the 5’UTR of *dmrt2a* until the 3’end of the DM domain, which contains the two DNA-binding domains and the crucial amino acid residues for nuclear translocation (Fig. 5c) [13]. We were able to identify only one ATG in *dmrt2a* mutants, located near the 3’end of the DM domain that maintained *dmrt2a* reading frame (Fig. 5c). If this ATG was used, the DM domain would be truncated and consequently, would lack the residues shown to be crucial for nuclear translocation. As Dmrt2a is a transcription factor, we would expect that the inability to enter the nucleus would render this protein dysfunctional. Regarding *dmrt2b* mutants, we were able to find an alternative ATG, after the premature STOP codon, which enabled the production of a functional DM domain (Fig. 5c). However, there is no guarantee that this ATG is in an appropriate genomic environment to allow its use.

In the absence of specific antibodies against Dmrt2a and Dmrt2b to evaluate the production of these proteins, we cannot be sure that we have true null mutants. Therefore, we used an indirect approach: we evaluated the levels of both transcripts based on the assumption that, if *dmrt2a* and *dmrt2b* transcripts are dysfunctional they should be degraded.

**Fig. 7:**
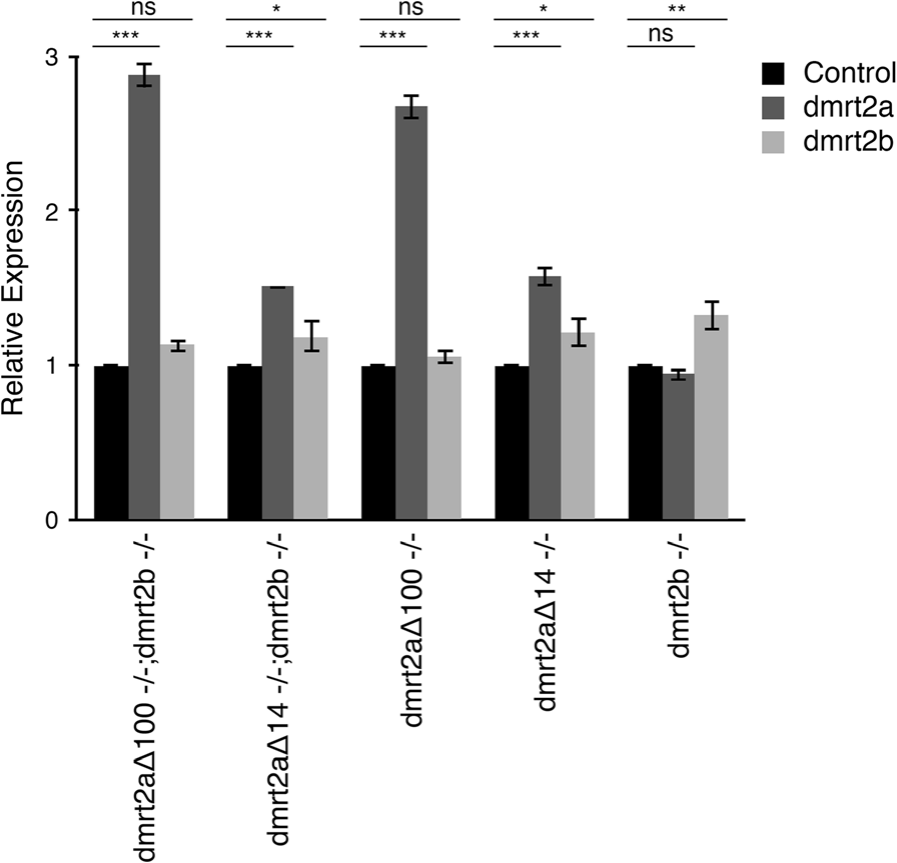
Evaluation of *dmrt2a* and *dmrt2b* transcript levels in single and double mutants at 24 hpf. *P < 0.05, **P < 0.01, ***P < 0.001, ns: not significant. *P*-values were generated using a two-tailed *t*-test. Hpf: hours post-fertilisation

The results we obtained from this evaluation were the following: (1) in single *dmrt2a* mutants and in double mutants, *dmrt2a* transcript was increased; (2) in single *dmrt2b* mutants, its transcript was increased and *dmrt2a* transcript was unaffected; (3) in single *dmrt2a* mutants and in double mutants, *dmrt2b* transcript was not affected (*dmrt2a*^*Δ100−/−*^ and *dmrt2a*^*Δ100−/−*^;*dmrt2b*^*−/−*^) or was only slightly affected (*dmrt2a*^*Δ14−/−*^ and *dmrt2a*^*Δ14−/−*^;*dmrt2b*^*−/−*^) (Fig. 7). These results show that *dmrt2a* and *dmrt2b* transcripts are under a negative feedback loop regulating their own expression, which suggests a deficiency in the production of Dmrt2a and Dmrt2b proteins. However, we still cannot assume that the mutations obtained produce null alleles. Our data also corroborates the lack of redundancy between *dmrt2a* and *dmrt2b*, further supporting previously published results [8].

### *dmrt2a*-MO proves to be specific and non-toxic

At this moment, we lack specific antibodies to confirm if the different mutants obtained are true nulls and, assuming that a compensatory mechanism is taking place, we have yet to identify possible candidate gene(s) for this role. Nevertheless, we took advantage of one of our mutant lines to re-validate *dmrt2a*-MO [3, 5].

**Fig. 8:**
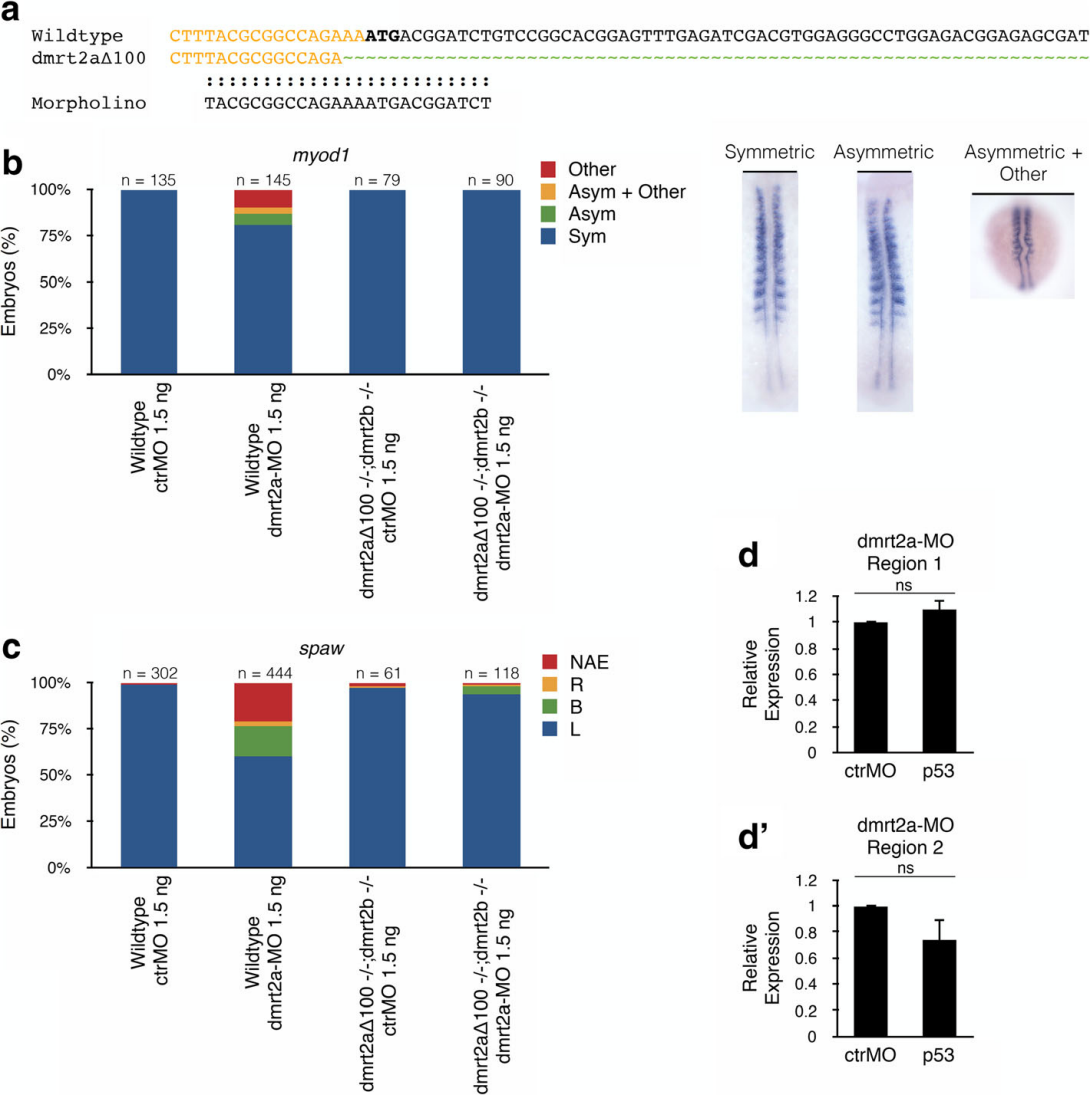
Evaluation of the specificity of *dmrt2a*-MO. **a** Alignment between wildtype *dmrt2a* sequence, *dmrt2a*^*Δ100*^ sequence and *dmrt2a*-MO. The MO is represented in the sense orientation. In bold caps is represented the start codon, in green the indels and in orange the 3’end of the 5’UTR. **b, c** Validation of the specificity of *dmrt2a*-MO using (**b**) *myod1* (14-somite stage) and (**c**) *spaw* (20-somite stage). The embryos shown illustrate the different phenotypes obtained in *myod1* expression. **d, d’** Evaluation of *p53* levels by qPCR in (**d**) Region 1 (somites) and (**d’**) Region 2 (tailbud/LRO) after injecting *dmrt2a*-MO in wildtype embryos. UTR: Untranslated Region, L: left, R: right, B: bilateral, NAE: no anterior expansion, Sym: symmetric, Asym: asymmetric, ctrMO: control morpholino, ns: not significant. *P*-values were generated using a two-tailed *t*-test

**Fig. 9:**
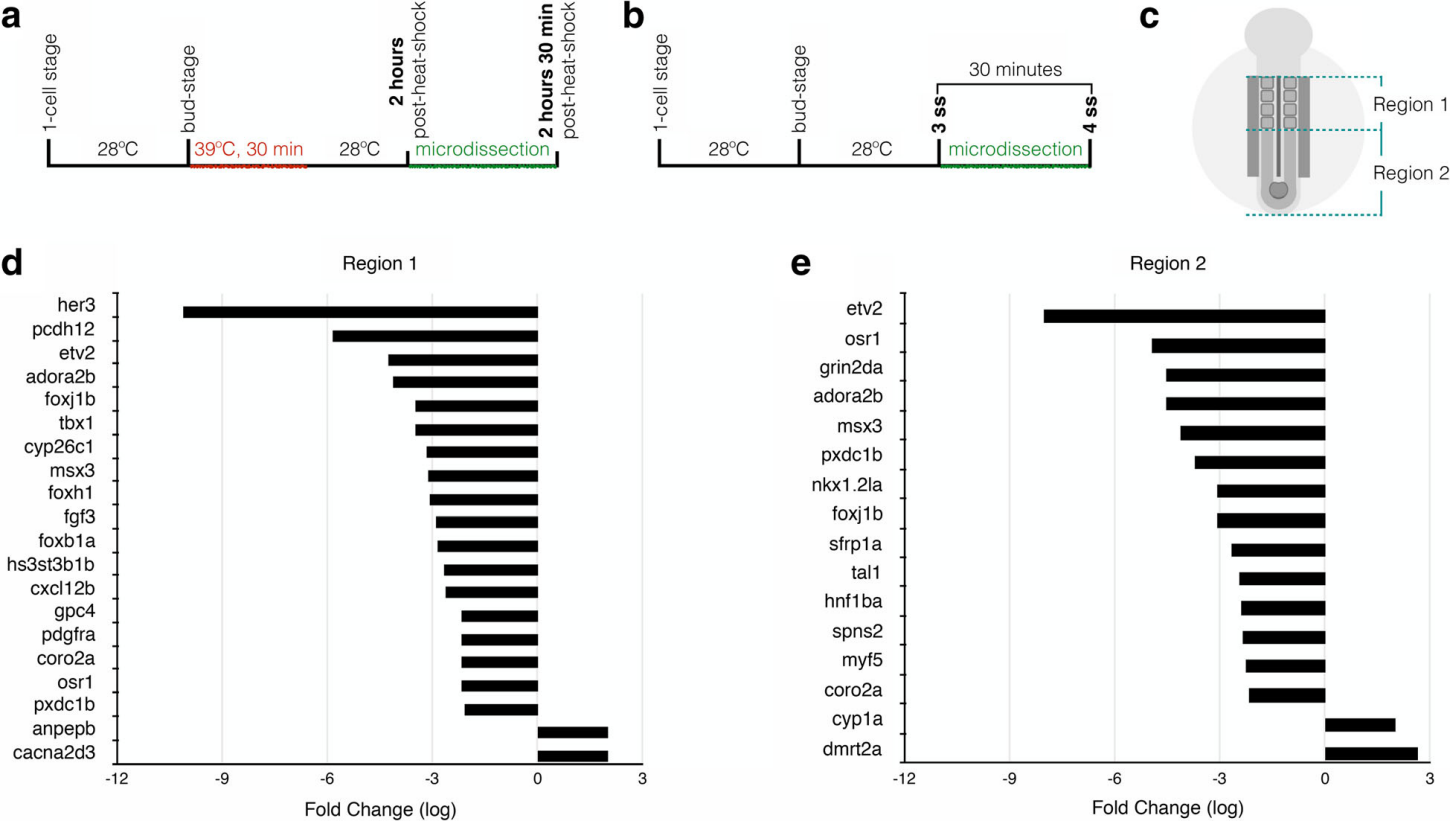
Microdissection protocols and microarray selected data. **a** Representation of the procedure used for the gain-of-function approaches (microarray and qPCR). **b** Representation of the procedure used for the loss-of-function approach. **c** Representation of the microdissected regions. **d** Selected genes from Region 1 microarray with FC > 2 or FC < − 2, *P* < 0.05 and FDR < 0.05. **e** Selected genes from Region 2 microarray with FC > 2 or FC < − 2, *P* < 0.05 and FDR < 0.05. min: minutes, ss: somite stage

To accomplish this, we used the *dmrt2a*^*Δ100−/−*^ background, which lacks 13 nucleotides of the *dmrt2a*-MO binding site (Fig. 8a). As the morpholino lacks it preferred binding site, it is the ideal situation to evaluate if it can bind non-specifically to other regions in the genome. To perform this evaluation, we injected *dmrt2a*-MO in the mutant background and compared it with wildtype embryos injected in the same conditions. After injecting *dmrt2a*-MO in a wildtype background, we observed the expected phenotype in *myod1* and *spaw* expression (Fig. 8b, c). In contrast, this phenotype was absent or extremely reduced when injecting *dmrt2a*-MO in the mutant background (Fig. 8b, c). Our results suggest that, in the evaluated conditions, *dmrt2a*-MO does not have detectable off-target effects. Additionally, we observed that the toxicity marker – *p53* – was not significantly affected after *dmrt2a*-MO injection (Figs. 8d, d’ and 9b, c for procedure details). As *dmrt2a*-MO seems to be specific, without off-targets and non-toxic, we decided to use it as a preferential loss-of-function tool.

### Genome wide approach to identify Dmrt2a downstream genes

In the context of the Tg(hsp70:HA-*dmrt2a*), the HA-Dmrt2a fusion protein peaks between 1 h 30 min and 2 h post-heat-shock (Additional file 2: Figure S2). Given that our goal was to identify Dmrt2a most immediate downstream genes we decided to collect embryo samples between 2 h (~ 3-somite stage) and 2 h 30 min (~ 4-somite stage) after heat-shock (Fig. 9a). Embryo samples were collected using a microdissection protocol. We microdissected Region 1 (containing somites) and Region 2 (containing tailbud/LRO) which are *dmrt2a* expressing territories (Fig. 9c). These samples were used in a microarray analysis.

In the microarray data (Fig. 9d, e and Additional file 3: Figure S3a, b), *dmrt2a* levels are increased as expected. However, *dmrt2a* is significantly increased in Region 2 (Fold change (FC) = 2.63, *P* = 0.000008, False Discovery Rate (FDR) = 0.0212) but not in Region 1 (FC = 1.3, *P* = 0.000416, FDR = 0.0677). This could be due to the higher physiological expression levels of *dmrt2a* in somites (Region 1) when compared to tailbud/LRO (Region 2).

DMRT proteins were described to act as activators, repressors or both [17, 18]. Our microarray analysis revealed that Dmrt2a seems to act mainly as a repressor during early development.

The analysis of the microarray showed some genes that could be expected to be downstream of Dmrt2a, such as *myf5* (myogenesis related [10]) and *foxj1b* (left-right asymmetry related [19]). Interestingly, a category of genes that was revealed to be potentially downstream of Dmrt2a, is related to cardiovascular development (Additional file 4: Figure S4a, b).

**Fig. 10:**
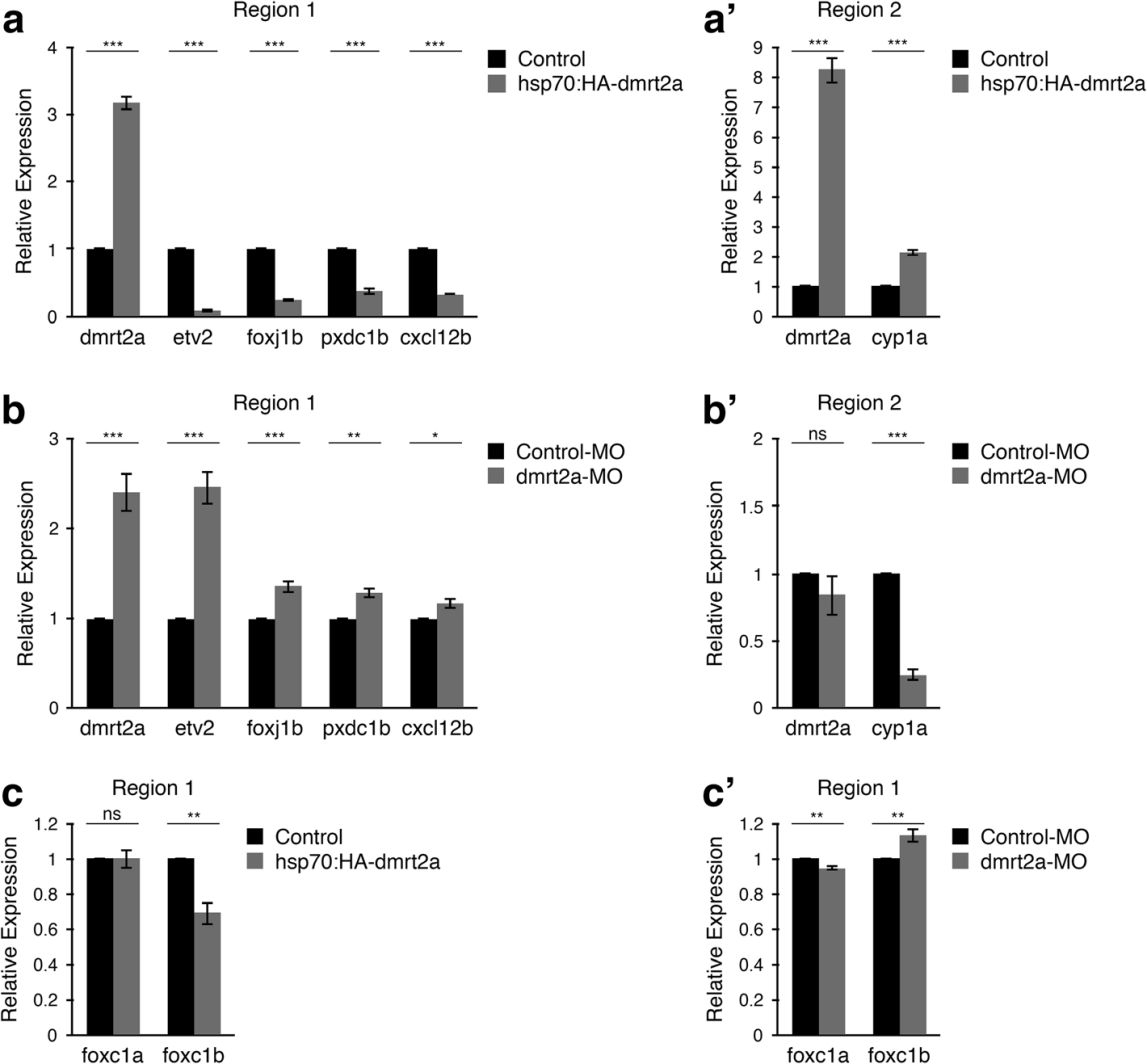
Microarray data validation. **a, a’** qPCR validation of selected genes from Region 1 (**a**) and Region 2 (**a’**), in a gain-of-function context. **b, b’** qPCR of selected genes from Region 1 (**b**) and Region 2 (**b’**), in a loss-of-function context. **c, c’** Evaluation of *foxc1a* and *foxc1b* transcripts by qPCR using Region 1 samples, in gain-of-function (**c**) and loss-of-function (**c’**) experiments. *P < 0.05, **P < 0.01, ***P < 0.001, ns: not significant. *P*-values were generated using a two-tailed *t*-test

We proceeded to validate the microarray data by qPCR. All the selected genes were independently validated, except for *ctslb* in Region 1 (Fig. 10a, a’ and Additional file 5: Figure S5a, a’).

### Validation of six genes downstream of Dmrt2a: *foxj1b*, *pxdc1b*, *cxcl12b*, *etv2*, *foxc1b* and *cyp1a*

To ensure that the selected genes were not a result of *dmrt2a* misexpression, we determined their level of expression in a knockdown context, using the previously validated *dmrt2a*-MO. In this combinatorial approach, only the genes down-regulated in the gain-of-function and up-regulated in the loss-of-function, or vice-versa, would be considered to be truly downstream of Dmrt2a activity.

In embryos injected with control-MO or *dmrt2a*-MO, we microdissected Region 1 and Region 2 (Fig. 9b, c) and did qPCR of the previously validated genes (Fig. 10b, b’ and Additional file 5: Figure S5b, b’).

Using this strategy, we were able to validate five genes: *foxj1b*, *pxdc1b, cxcl12b, etv2* (Region 1) and *cyp1a* (Region 2) (Fig. 10a-b’, for an illustration of the expression pattern of these genes see Additional file 6: Figure S6A1-J2, and for *dmrt2a* expression pattern see Additional file 7: Figure S7A1-B2’). To ensure that the change of the expression levels of the five selected genes was not due to *dmrt2a*-MO off-target effects, we injected this MO in the mutant background and compared it with wildtype embryos. We analysed these embryos using in situ hybridisation and showed that these genes are specifically affected by *dmrt2a*-MO (Additional file 7: Figure S7C1-C3’ and Additional file 8: Figure S8A1-E3). We also observed that similarly to what happens in *dmrt2a* mutants, in morphant embryos, *dmrt2a* transcripts are also up-regulated (Figs. 7 and 10b, Additional file 5: Figure S5b and Additional file 7: Figure S7A1-A2’).

As Etv2 is a transcription factor with a well-described interaction with the Foxc proteins [20, 21], we verified the presence or absence of Foxc proteins in the microarray data. We found that the zebrafish homolog genes of the mouse *Foxc1* – *foxc1a* and *foxc1b* – were present in the microarray data but with low fold changes (FC = − 1.04, *P* = 0.219, FDR = 0.636; FC = − 1.3, *P* = 0.00134, FDR = 0.103, respectively) (Additional file 3: Figure S3a). While *foxc1a* could not be validated by the loss-of-function approach, we verified that *foxc1b* was indeed down-regulated after *dmrt2a* overexpression and up-regulated after *dmrt2a* loss-of-function (Fig. 10c, c’, for an illustration of the expression pattern of *foxc1b* see Additional file 6: Figure S6 K1-L2’). To ensure that the change of the expression levels of *foxc1b* was not due to *dmrt2a*-MO off-target effects, we injected this MO in the mutant background, as before. We analysed these embryos using in situ hybridisation and showed that *foxc1b* is specifically affected by *dmrt2a*-MO (Additional file 8: Figure S8F1-F3’).

In this work, we identified and validated six genes downstream of Dmrt2a during early development: *foxj1b*, *pxdc1b, cxcl12b, etv2*, *foxc1b* and *cyp1a*. The relationship between them and Dmrt2a, and their contribution to the different Dmrt2a functions, will be the subject of future studies.

## Discussion

In order to establish the mechanism of action of Dmrt2a, one needs to identify its downstream genes. In this work, we generated and validated several genetic tools that allowed us to identify six genes downstream of Dmrt2a during early embryonic development: *foxj1b*, *pxdc1b, cxcl12b, etv2*, *foxc1b* and *cyp1a*.

To develop one of our genetic tools – stable *dmrt2a* mutant lines – we used the TALEN technology. However, when considering the different *dmrt2a* mutant lines we generated, we were surprised to detect only a very weak or negligible phenotype, when compared to the previously published morpholino data [3, 5, 8]. The robustness of *dmrt2a* knockdown phenotype had been previously established in two independent studies [3, 5], especially in the work of Matsui et al. [5], and its specificity was further confirmed with our work.

The weak phenotype obtained could not be due to the maternal contribution of *dmrt2a* transcripts because we always used an incross between homozygous mutant fish. The presence of alternative ATGs could be an issue, as discussed before, mainly in the case of *dmrt2b* mutant. Another possibility for the weak mutant phenotype is the presence of compensatory gene(s). Our data indicate that *dmrt2b* does not seem to compensate for the lack of *dmrt2a*, supporting the findings of Liu et al. [8]*.* Therefore, to uncover if a compensatory mechanism is taking place, a genome wide approach could be performed comparing mutants and morphants.

To prove the absence of mutant proteins we need specific antibodies that are not available. Thus, in an attempt to understand the outcome of our mutations, we evaluated *dmrt2a* and *dmrt2b* transcripts in the different mutant backgrounds. In theory, if the transcripts are not functional they should be degraded. This hypothesis should have been true at least in the case of *dmrt2b* mutant, due to non-sense mediated decay. However, we observed that *dmrt2a* and *dmrt2b* transcripts are up-regulated in the different mutants evaluated. This suggests that, in the absence of a completely functional transcript/protein, the stimulus to constantly produce more transcript remains, further suggesting that Dmrt2a and Dmrt2b proteins, if produced, should not be completely functional. This does not exclude the possibility of degradation of the mutant transcripts, only that the rate of transcript degradation is slower than the rate of production.

Regarding future approaches to this problem, an effort should be placed in the identification of *dmrt2a* and *dmrt2b* promoter regions, in the generation of bona fide null mutants and in the production of specific antibodies.

As we have no certainty that *dmrt2a* mutants are nulls, we decided against using these lines in the microarray approach. Yet, we were able to take advantage of them to validate *dmrt2a*-MO, confirming its specificity. Therefore, we performed the microarray with Tg(hsp70:HA-*dmrt2a*) and validated its data using *dmrt2a*-MO.

From the list of microarray validated genes, *foxj1b* [19] is a strong candidate to mediate the left-right asymmetry phenotypes of Dmrt2a, which were previously described in gain and loss-of-function approaches.

With our timely controlled *dmrt2a* overexpression experiments we identified a new phenotype in somite border formation, which recalls the *Dmrt2* mouse mutant phenotype [9]. The defects observed in the mouse mutants were associated with a strong reduction of the levels of LAMININ-1, an extracellular matrix protein [9]. While in our pool of six validated genes, we did not identify classic extracellular matrix related genes, we found an interesting candidate, *pxdc1b*. Pxdc1b belongs to a family related to membrane attachment to organelles of the endocytic and secretory systems via binding of phosphoinositide lipids [22]. As extracellular matrix proteins are continuously being secreted [23], *pxdc1b* could establish a link between Dmrt2a, extracellular matrix deposition and somite border defects.

Additionally, in our microarray, we identified *cyp1a* as a gene downstream of Dmrt2a. This cytochrome was one of the few genes up-regulated after *dmrt2a* overexpression and the only one that was validated by the loss-of-function approach. Also, *cyp1a* is the only validated gene from the microdissected Region 2. However, Cyp1a physiological function in the embryo remains unknown therefore, its interaction with Dmrt2a will require further analysis.

Interestingly, we validated three genes related to vascular development: *cxcl12b, etv2* and *foxc1b*. Although we lack functional data to explain the relationship between Dmrt2a and these genes, when considering *dmrt2a* expression pattern within the somite, we can contemplate the hypothesis that Dmrt2a is affecting the small population of somite-derived endothelial cells [24, 25].

*cxcl12b* is expressed in a subset of the myotome, named endotome, which will colonise the dorsal aorta and will contribute to endothelial and vascular associated cells in zebrafish [25]. As *dmrt2a* is also expressed in the myotome [2], and as it acts as a transcriptional repressor, we can speculate that it could restrict *cxcl12b* endotome expression domain.

*etv2*-expressing angioblasts (endothelial cells precursors) migrate from the lateral plate mesoderm, intersomitically, towards the midline, where they contribute to the dorsal aorta and posterior cardinal vein [26]. Therefore, a possible cell non-autonomous interaction between Dmrt2a and *etv2* could occur at somite territory.

Foxc proteins have been previously described to interact with *etv2* [21] thus, it would be worthwhile to analyse if, the somite-expressed gene *foxc1b*, can cooperate with *etv2* downstream of Dmrt2a. Moreover in mouse, CXCL12 receptor – CXCR4 – is transcriptionally activated by FOXC1 and FOXC2 in endothelial cells [27]. Although in zebrafish, a link between Cxcl12 signalling and Foxc transcription factors has not been established, it would be interesting to evaluate if this interaction could occur.

## Conclusions

In this study, we generated and characterised an inducible *dmrt2a* transgenic line and mutant lines for *dmrt2a* and *dmrt2b*. Using our transgenic line we described for the first time *dmrt2a* overexpression phenotype. We confirmed that the mild phenotype present in *dmrt2a* mutant lines is not due to a compensatory mechanism mediated by *dmrt2b*. Additionally, we took advantage of one of our *dmrt2a* mutant lines to confirm *dmrt2a*-MO specificity, validating this tool.

Using our tools we performed and validated a microarray experiment that allowed the identification of six genes downstream of Dmrt2a: *foxj1b*, *pxdc1b, cxcl12b, etv2*, *foxc1b* and *cyp1a*.

The identification of these six genes will contribute to the understanding of Dmrt2a mechanism of action during zebrafish early development.

In which embryonic domains is Dmrt2a controlling these genes, if their interaction with Dmrt2a is direct or not and, how can these genes contribute to the previously described Dmrt2a phenotypes, will be addressed in future studies.

## Methods

### Zebrafish lines maintenance and husbandry

All zebrafish (*Danio rerio*) lines used had Tubingen background and were maintained at 28 °C on a 14-h light/10-h dark cycle. The wildtype fish used to generate the transgenic and mutant lines were obtained from Zebrafish International Resource Center (ZIRC). Adult zebrafish were only used as breeders. The embryos when collected were kept in an incubator at 28 °C, under the same light/dark cycle, until the appropriate stage of development [11].

When growing a new fish line, embryos were bleached before reaching 24 hpf, as described [28]. After 5 days in the incubator at 28 °C, 40–50 larvae were transferred to tanks of 3 l, where they grew during 1 month. The number of juvenile fish per litre decreased gradually until reaching adulthood. Adult breeding zebrafish (3 months old) were maintained at a density of five fish per litre.

Zebrafish were raised and maintained in a six-rack recirculating system from Tecniplast supplied by a reverse osmosis unit. Conductivity was maintained at 800 μS with ocean salt (Aquarium Systems) and pH was maintained at 7.0, and adjusted with sodium bicarbonate (Sigma-Aldrich). Fish were fed with ZEBRAFEED (Sparos, Portugal) < 100 μm (larvae), between 100 and 200 μm (juvenile fish) and between 200 and 400 μm (adults). Additionally, adult fish ate a meal of decapsulated *Artemia* (ZM Systems) per day.

All experiments were performed with embryos obtained from an outcross of homozygous progenitors, in the case of transgenic fish, and from an incross of two homozygous progenitors, in the case of mutant fish.

### Transgenic line generation

To generate the Tg(hsp70:HA-*dmrt2a*), *dmrt2a* cDNA with an N-terminal HA-tag was placed downstream of the *hsp70* heat-shock promoter in the pT2 vector (UAS-hsp70 promoter-polyA-γ-crystallin promoter-CFP). *dmrt2a* total RNA was extracted using TRIzol (Thermo Fisher), and cDNA was synthesised using the M-MLV Reverse Transcriptase Kit (Thermo Fisher). The transgenic construct was generated using a set of PCR reactions: (1) *dmrt2a* sequence was amplified using the pF-dmrt2a-HA-Nter (5’-CCAGACTACGCTTCCCTTATGACGGATCTGTCCGGCACGGAG-3′) to add the C-terminal of the HA sequence to the N-terminal of *dmrt2a* and a pR-dmrt2a-StuI (5’-AGGGGAAAACTGAGATTTCCGATTTAAAGAAAGCGC-3′) to add a *Stu*I restriction site to the C-terminal of *dmrt2a* and, (2) using the primer pF-HA-K-ClaI (5’-CCATCGATGGCCACCATGGCTTCATATCCTTACGATG-3′) we generated the full-length HA sequence, added the Kozak sequence and the *Cla*I restriction site. This construct was cloned into the pGEM-T Easy Vector (Promega) and screened using the blue-white screening technique. Next, this construct and the pT2 vector were digested with *Cla*I and *Stu*I (New England BioLabs) and gel purified using the Wizard SV Gel and PCR Clean-Up System (Promega). A ligation reaction between the *Cla*I-Kozak-HA-*dmrt2a*-*Stu*I fragment and the pT2 vector was performed using the T4 ligase (Promega). The positive colonies were selected and the DNA purified through a Genopure Plasmid Midi Kit (Roche). 20 pg (14 ng.μL^− 1^) of pT2 plasmid containing the hsp70:HA-*dmrt2a* insert were injected into one-cell stage zebrafish embryos together with 7 pg (5 ng.μL^− 1^) of transposase mRNA.

### Southern blot

Southern blot analysis was performed as described [29]. Briefly, F1 heterozygous adult transgenic fish were anaesthetised using Tricaine (Sigma-Aldrich). The tail fin was cut, and genomic DNA was extracted. About 10 μg of DNA were digested with *Stu*I (New England Biolabs), and the DNA fragments were separated by gel electrophoresis, at 30 V overnight. Afterwards, the gel was stained and washed, and the DNA transferred to a nylon membrane positively charged (Roche). To cross-link the DNA, the membrane was irradiated using UV light (λ = 254 nm). The pre-hybridisation and hybridisation were performed using the ULTRAhyb buffer (Ambion). The radiolabelled probe was added at 10^9^ cpm.mL^− 1^ of ULTRAhyb, and the hybridisation was carried out at 42 °C for 16–24 h. The γ-crystallin probe was amplified from the pT2 vector using the set of primers, pFCrisSB1 (5’-ATACGACACTGCATGGATCACCTGAAAG-3′) and pRCrisSB1 (5’-CTTTACCCAAAGAGTTATCCAGCATTCC-3′), which generated a fragment of 561 bp. The PCR product was gel purified using the Wizard SV Gel and PCR Clean-Up System (Promega). The DNA was labelled with ^32^P-dATP (PerkinElmer) using the Random Primer Labelling Kit (Stratagene). The signal was detected using the Phosphoimager Cassette (Molecular Dynamics) and analysed using the Typhoon System Model 9210 (GE Healthcare Life Sciences).

### Heat-shock experiments

In all heat-shock experiments, embryos were raised at 28 °C. At bud-stage, embryos were placed in embryo medium (5.02 mM NaCl, 0.17 mM KCl, 0.33 mM CaCl_2_.2H_2_O, 0.33 mM MgSO_4_.7H_2_O, 10^− 5^% methylene blue) pre-warmed at 39 °C, and heat-shocked for 30 min at the same temperature. Embryos were then collected and maintained at 28 °C until the appropriate stage of development. Tg(hsp70:HA-*dmrt2a*) and control embryos were heat-shocked with the same procedure.

### Genome editing

TALEN were designed using TAL Effector-Nucleotide Targeter (TALE-NT) 2.0 web based tools [30] together with the R-based tool developed by Jorge Velez (NICHD). The selected genomic regions for *dmrt2a* and *dmrt2b* were introduced in TALEN Targeter. Custom Spacer/Repeat Variable Diresidue (RVD) Lengths were used, with the spacer ranging from 14 to 17 nucleotides and the RVD lengths ranging from 16 to 21 nucleotides. The G substitute chosen was NN. The upstream base chosen to each monomer was T.

For *dmrt2a,* we designed one TALEN pair with a spacer containing the ATG start codon, Pair-1 (P_1_) (**TCTTGCTTTACGCGGCCAGAAAATGACGGATCTGTCCGG** start codon and the DM GCACGGAGTTTGAGA), and another TALEN pair further downstream, Pair-2 (P_2_) (**TCAGGCGGTGTTCACCGGCCCC**GGAGAGGACGACAC**GGGGTCCAAAGACGACGACAAA**). For *dmrt2b,* we designed a TALEN pair targeting the region between the ATG start codon and the DM domain, Pair-3 (P_3_) (**TGCGACCCACCTAGAGTGCC**TGAAAGCGGAGTGGG**TTGCAGACTGCGCCGGACGGGA**). TALEN binding sites are in bold caps, and the spacer region is underlined. Paired Target Finder was used to check for off-targets, the Score Cut-off chosen was 3.0.

To generate the single *dmrt2a* mutants (*dmrt2a*^*Δ100*^ and *dmrt2a*^*Δ14*^), 200 pg of P_1_ and 200 pg of P_2_ RNA were co-injected into one-cell stage embryos. To generate the single *dmrt2b* mutants, 250 pg of P_3_ RNA was injected into one-cell stage embryos. RNA was synthesised using mMESSAGE mMACHINE SP6 Transcription Kit (Thermo Fisher). To generate the double mutants, *dmrt2a*^*Δ100−/−*^ was crossed with *dmrt2b*^*−/−*^ and *dmrt2a*^*Δ14−/−*^ was crossed with *dmrt2b*^*−/−*^.

To screen the F0 mosaics and the following generations of *dmrt2b* single and double mutants, a High Resolution Melting screening method was developed using Corbett Rotorgene 6000 (Corbett Life Science) and Power SYBR Green PCR Master Mix (Thermo Fisher). The primers used for this method were: pF-HRMdmrt2b (5’-CACAGGTAGATGCGACCCAC-3′) and pR-HRMdmrt2b (5’-CTTCATCCGTGCCCATGACC-3′). To screen *dmrt2a*^*Δ100*^ and *dmrt2a*^*Δ14*^ single and double mutants, a PCR followed by gel electrophoresis was used with the following primers: pFdmrt2a (5’-GACACGTTACATGCAGGAAAACA-3′) and pRdmrt2a (5’-GCGGTCAGATTTGTCGTCGT-3′).

### Morpholino microinjection

A *dmrt2a* antisense morpholino targeting the ATG region (5’-AGATCCGTCATTTTCTGGCCGCGTA-3′) [3, 5] and a standard control morpholino (5’-CCTCTTACCTCAGTTACAATTTATA-3′) obtained from Gene Tools, were injected at 1.5 ng/embryo, into one-cell stage embryos. In the morpholino validation experiments, we injected *dmrt2a* morpholino in the mutant background, in the same days and using the same settings as in the wildtype counterparts, to ensure a controlled procedure.

### In situ hybridisation and immunohistochemistry

Single whole-mount in situ hybridisation was performed as described [31]. Embryos were photographed with a LEICA Z6 APO stereoscope coupled to a LEICA DFC490 camera.

Digoxigenin-labelled antisense RNA probes were synthesised from DNA templates of *deltaC* [32], *her1* [33], *her7* [34], *mespb* [35], *pcdh8* [36], *myod1* [37], *cb1045* [38], *spaw* [39], *pitx2* [40], *myl7* [41], *cxcl12b* [25], *etv2* [42], *foxj1b* [19], *foxc1b* [43], *dmrt2a* [4], *cyp1a,* which was cloned in pGEM-T Easy (Promega) using the pF-cyp1a (5’-ATGGCTCTGACTATTCTTCCAATATTGGG-3′) and pR-cyp1a (5’-CTAGAACCCAGGCTGTGGTGTGACCCGA-3′) and *pxdc1b,* which was cloned in pGEM-T Easy (Promega) using the pF-pxdc1b (5’-ATGGCATCGGCGATTTTTGAGGGCA-3′) and pR-pxdc1b (5’-AAGTCAGTTTCAAAAGGAACCAGA-3′).

For HA immunohistochemistry, the embryos were fixed after heat-shock in 4% paraformaldehyde overnight, incubated with rat anti-HA antibody (1:200, 3F10, Roche #11867423001) followed by anti-rat Alexa Fluor 594 (1:500, Thermo Fisher #A11007). F-actin and nuclei were detected with Alexa Fluor 488-Phalloidin (1:400, Thermo Fisher #A12379) and DAPI (10 μL.mL^− 1^), respectively. Embryos were photographed with a Zeiss LSM 510 Meta.

### Western blot

Embryos were collected 1 h 30 min and 2 h 30 min after heat-shock, treated as described [44] and kept at − 70 °C. The following procedure was performed as described [45]. For HA-tag detection, the membranes were incubated overnight with rabbit anti-HA antibody (1:1000, Y^− 11^, sc-805, Santa Cruz). For beta-actin, we used a rabbit anti-beta-actin (1:5000, ab8227, Abcam). As secondary antibody, we used an HRP-conjugated mouse anti-rabbit IgG antibody (1:10000, sc-2357, Santa Cruz).

### Microarray

Between the period of 2 h and 2 h 30 min after heat-shock, Tg(hsp70:HA-*dmrt2a*) and wildtype embryos were microdissected as described [46]. Tissues from Region 1 and Region 2 (Fig. 9a, c) were kept in TRIzol (Thermo Fisher) at − 70 °C. We obtained a pool of 100 pieces from Region 1 and 100 pieces from Region 2, from each condition, per replicate. Three biological replicates for each condition were used. Total RNA was extracted using TRIzol (Thermo Fisher) and further purified with RNA Clean & Concentrator-5 kit (Zymo Research). RNA quality was assessed with an Agilent 2100 Bioanalyzer (Agilent Genomics), and all samples had a RIN value between 8.60 and 9.30. All samples were analysed with Affymetrix Zebrafish Gene 1.1 ST Array Strip (Thermo Fisher). The raw data (CEL files) were analysed with Affymetrix Expression Console Software, Affymetrix Transcriptome Analysis Console Software (Thermo Fisher) and R. Statistical analysis was performed using one-way ANOVA and False Discovery Rate (FDR) correction. Only genes with a fold change (FC) higher than 2 or lower than − 2, with *P* < 0.05 and FDR < 0.05, were considered with the exception of *foxc1b* with a FC = − 1,3, *P* = 0.00134 and FDR = 0.103. Gene Ontology (GO) analysis was performed using AmiGO [47] with Bonferroni correction.

### Quantitative RT-PCR

Total RNA was extracted from zebrafish embryos with the appropriate stage, using TRIzol (Thermo Fisher) and further purified using the Direct-zol RNA Miniprep Kit (Zymo Research). For the evaluation of *dmrt2a* transcript throughout development, we used *n* = 30 wildtype embryos, per replicate. For the gain-of-function experiment, we collected embryos from the Tg(hsp70:HA-*dmrt2a*) line and wildtype embryos, and performed a heat-shock (Fig. 9a). All embryos were microdissected in Region 1 and Region 2 (Fig. 9a, c). From this approach, we obtained a pool of 60 pieces from Region 1 and a pool of 60 pieces from Region 2, from each condition (experimental and control), per replicate. For the loss-of-function experiment, we injected wildtype embryos with *dmrt2a*-MO or control-MO, and microdissected them in Region 1 and Region 2 (Fig. 9b, c). From this approach, we obtained a pool of 46 pieces from Region 1 and a pool of 46 pieces from Region 2, from each condition (experimental and control), per replicate. For the evaluation of mutant transcripts, we used the different *dmrt2a* and *dmrt2b* mutant embryos and wildtype embryos, at 24 hpf, with *n* = 10, per replicate. Three biological replicates were used in each experiment. cDNA was synthesised using DyNAmo cDNA Synthesis Kit (Thermo Scientific) using random hexamer primers. Quantitative PCR was performed using Corbett Rotorgene 6000 (Corbett Life Science) and Power SYBR Green PCR Master Mix (Thermo Fisher). Quantification of the relative expression was performed using the 2^-ΔΔCt^ method. We evaluated each gene on an individual basis, between experimental and control samples. Data were analysed using a two-tailed *t*-test. The primers used are listed in Additional file 9: Table S1.

### Statistical analysis

All experiments were performed with at least three biological replicates, collected from different breeders. The statistical analysis performed in the microarray experiment was a one-way ANOVA and in qPCR experiments was a two-tailed *t*-test. *P* values of less than 0.05 were considered significant.

## Additional files

Additional file 1: **Figure S1.** TALEN pairs. **a** Alignment between TALEN P_1_ and *dmrt2a* sequence. **b** Alignment between TALEN P_2_ and *dmrt2a* sequence. **c** Alignment between TALEN P_3_ and *dmrt2b* sequence. The start codon is underlined, and the spacer region is in lowercase. Each colour represents a different Repeat Variable Diresidue (RVD). (TIF 3783 kb)

Additional file 2: **Figure S2.** HA-Dmrt2a dynamics after heat-shock. Immunostaining showing the expression of the HA-Dmrt2a fusion protein at different time-points post-heat-shock. The HA-Dmrt2a protein is depicted in red, and the nucleus is depicted in blue. Min: minutes. Scale bar: 10 μm. (TIF 16094 kb)

Additional file 3: **Figure S3.** Microarray data. **a, b** List of all the genes obtained in the microarray with FC > 2 or FC < − 2, *P* < 0.05 and FDR < 0.05, together with *dmrt2a*, *foxc1a* and *foxc1b* in Region 1 (FC = 1.3, *P* = 0.000416, FDR = 0.0677; FC = − 1.04, *P* = 0.219, FDR = 0.636; FC = − 1.3, *P* = 0.00134, FDR = 0.103, respectively). (**a**) Region 1 and (**b**) Region 2. Genes related to cardiac and vascular development are underlined. (TIF 679 kb)

Additional file 4: **Figure S4.** Gene Ontology (GO) analysis of the microarray data. **a, b** Most significant GO terms associated with the microarray data with FC > 2 or FC < − 2, *P* < 0.05 and FDR < 0.05. (**a**) Region 1 and (**b**) Region 2. (TIF 1345 kb)

Additional file 5: **Figure S5.** qPCR validation of the microarray data in gain and loss-of-function experiments. **a, a’** qPCR validation of selected genes from the microarray in a gain-of-function approach. (**a**) Region 1 and (**a’**) Region 2. **b, b’** qPCR validation of selected genes from the microarray in a loss-of-function approach using *dmrt2a*-MO. (**b**) Region 1 and (**b’**) Region 2. *P < 0.05, **P < 0.01, ***P < 0.001, ns: not significant. *P*-values were generated using a two-tailed *t*-test. (TIF 1349 kb)

Additional file 6: **Figure S6.** No misexpression was found in the validated genes after *dmrt2a* gain and loss-of-function experiments. **A1-L2’** In situ hybridisation results depicting the six genes validated in this work. **A1-B2”** *etv2* expression pattern after injecting ctrMO (**A1-A1”**), *dmrt2a*-MO (**A2-A2”**), and after heat-shock in control (**B1-B1”**) and Tg(hsp70:HA-*dmrt2a*) (**B2-B2”**). **C1-D2”’** *foxj1b* expression pattern after injecting ctrMO (**C1-C1”’**), *dmrt2a*-MO (**C2-C2”’**), and after heat-shock in control (**D1-D1”’**) and Tg(hsp70:HA-*dmrt2a*) (**D2-D2”’**). **E1-F2”** *cyp1a* expression pattern after injecting ctrMO (**E1-E1”**), *dmrt2a*-MO (**E2-E2”**), and after heat-shock in control (**F1-F1”**) and Tg(hsp70:HA-*dmrt2a*) (**F2-F2”**). **G1-H2** *cxcl12b* expression pattern after injecting ctrMO (**G1**), *dmrt2a*-MO (**G2**), and after heat-shock in control (**H1**) and Tg(hsp70:HA-*dmrt2a*) (**H2**). **I1-J2** *pxdc1b* expression pattern after injecting ctrMO (**I1**), *dmrt2a*-MO (**I2**), and after heat-shock in control (**J1**) and Tg(hsp70:HA-*dmrt2a*) (**J2**). **K1-L2’** *foxc1b* expression pattern after injecting ctrMO (**K1, K1’**), *dmrt2a*-MO (**K2, K2’**), and after heat-shock in control (**L1, L1’**) and Tg(hsp70:HA-*dmrt2a*) (**L2, L2’**). After *dmrt2a* overexpression using Tg(hsp70:HA-*dmrt2a*) we did not observe misexpression of the six validated genes. Upon comparison with *dmrt2a*-MO injected embryos, we observed changes in the expression levels of some genes (arrowheads), according to qPCR data. As depicted with asterisks, after *dmrt2a*-MO injection we observed only very subtle changes in the expression pattern of some genes, corresponding to the less affected genes, as quantified by qPCR. All embryos were collected between 3 and 4-somite stage (loss-of-function experiments) and between 2 h and 2 h 30 min after heat-shock (gain-of-function experiments). (A1-B2, C1-D2, E1-F2, G1-H2, I1-J2, K1-L2) Lateral view, anterior to the left. (A1’-B2’, C1’-D2’, E1’-F2’) Dorsal-anterior view, anterior to the top. (A1”-B2”, C1”’-D2”’, E1”-F2”, K1’-L2’) Dorsal-posterior view, anterior to the top. (C1”-D2”) Dorsal-medial view, anterior to the top. ctrMO: control morpholino. (TIF 13209 kb)

Additional file 7: **Figure S7.** *dmrt2a* expression pattern using in situ hybridisation after gain and loss-of-function experiments. **A1-B2’** *dmrt2a* expression pattern after injecting ctrMO (**A1, A1’**), *dmrt2a*-MO (**A2, A2’**), and after heat-shock in control (**B1, B1’**) and Tg(hsp70:HA-*dmrt2a*) (**B2, B2’**). **C1-C3’** *dmrt2a* expression pattern in wildtype embryos (**C1, C1’**), in *dmrt2a*^*Δ100−/−*^*;dmrt2b*^*−/−*^ embryos injected with ctrMO (**C2, C2’**) and *dmrt2a*-MO (**C3, C3’**). (A1-C3) Lateral view, anterior to the left. (A1’-C3’) Dorsal view, anterior to the top. All embryos were collected between 3 and 4-somite stage (loss-of-function experiments) and between 2 h and 2 h 30 min after heat-shock (gain-of-function experiments). ctrMO: control morpholino, WT: wildtype. (TIF 3087 kb)

Additional file 8: **Figure S8.** *dmrt2a*-MO is specific for the validated genes. **A1-F3’** In situ hybridisation results depicting the six genes validated in this work. **A1-A3”** *etv2* expression pattern in wildtype embryos (**A1-A1”**), in *dmrt2a*^*Δ100−/−*^*;dmrt2b*^*−/−*^ embryos injected with ctrMO (**A2-A2”**) and *dmrt2a*-MO (**A3-A3”**). **B1-B3”’** *foxj1b* expression pattern in wildtype embryos (**B1-B1”’**), in *dmrt2a*^*Δ100−/−*^*;dmrt2b*^*−/−*^ embryos injected with ctrMO (**B2-B2”’**) and *dmrt2a*-MO (**B3-B3”’**). **C1-C3’** *cyp1a* expression pattern in wildtype embryos (**C1, C1’**), in *dmrt2a*^*Δ100−/−*^*;dmrt2b*^*−/−*^ embryos injected with ctrMO (**C2, C2’**) and *dmrt2a*-MO (**C3, C3’**). **D1-D3** *cxcl12b* expression pattern in wildtype embryos (**D1**), in *dmrt2a*^*Δ100−/−*^*;dmrt2b*^*−/−*^ embryos injected with ctrMO (**D2**) and *dmrt2a*-MO (**D3**). **E1-E3** *pxdc1b* expression pattern in wildtype embryos (**E1**), in *dmrt2a*^*Δ100−/−*^*;dmrt2b*^*−/−*^ embryos injected with ctrMO (**E2**) and *dmrt2a*-MO (**E3**). **F1-F3’** *foxc1b* expression pattern in wildtype embryos (**F1, F1’**), in *dmrt2a*^*Δ100−/−*^*;dmrt2b*^*−/−*^ embryos injected with ctrMO (**F2, F2’**) and *dmrt2a*-MO (**F3, F3’**). We did not observe obvious differences between the three different conditions evaluated. All embryos were collected between 3 and 4-somite stage. (A1-A3, B1-B3, C1-C3, D1-D3, E1-E3, F1-F3) Lateral view, anterior to the left. (A1’-A3’, B1’-B3’) Dorsal-anterior view, anterior to the top. (A1”-A3”, B1”’-B3”’, C1’-C3’, F1’-F3’) Dorsal-posterior view, anterior to the top. (B1”-B3”) Dorsal-medial view, anterior to the top. ctrMO: control morpholino, WT: wildtype. (TIF 8561 kb)

Additional file 9: **Table S1.** Primers used in quantitative RT-PCR. (DOC 97 kb)

## Abbreviations

cDNA: Complementary DNA
ctr: Control
DM: Doublesex and Mab3
DMRT: Doublesex and Mab3 related transcription factors
FC: Fold change
FDR: False discovery rate
GO: Gene ontology
HA: Haemagglutinin
hpf: Hours post-fertilisation
LRO: Left-Right Organiser
MO: Morpholino
mRNA: Messenger RNA
ns: Not significant
P_1_: TALEN pair 1
P_2_: TALEN pair 2
P_3_: TALEN pair 3
RIN: RNA integrity number
RVD: Repeat variable diresidue
TALEN: Transcription activator-like effector nucleases
UTR: Untranslated Region
WT: Wildtype
