# Supplementary figures and images for: Identification of Dmrt2a downstream genes during zebrafish early development using a timely controlled approach
Source: BMC Dev Biol. 2018 Jun 19;18:14. doi: 10.1186/s12861-018-0173-5 (PMC6006574; doi:10.1186/s12861-018-0173-5)

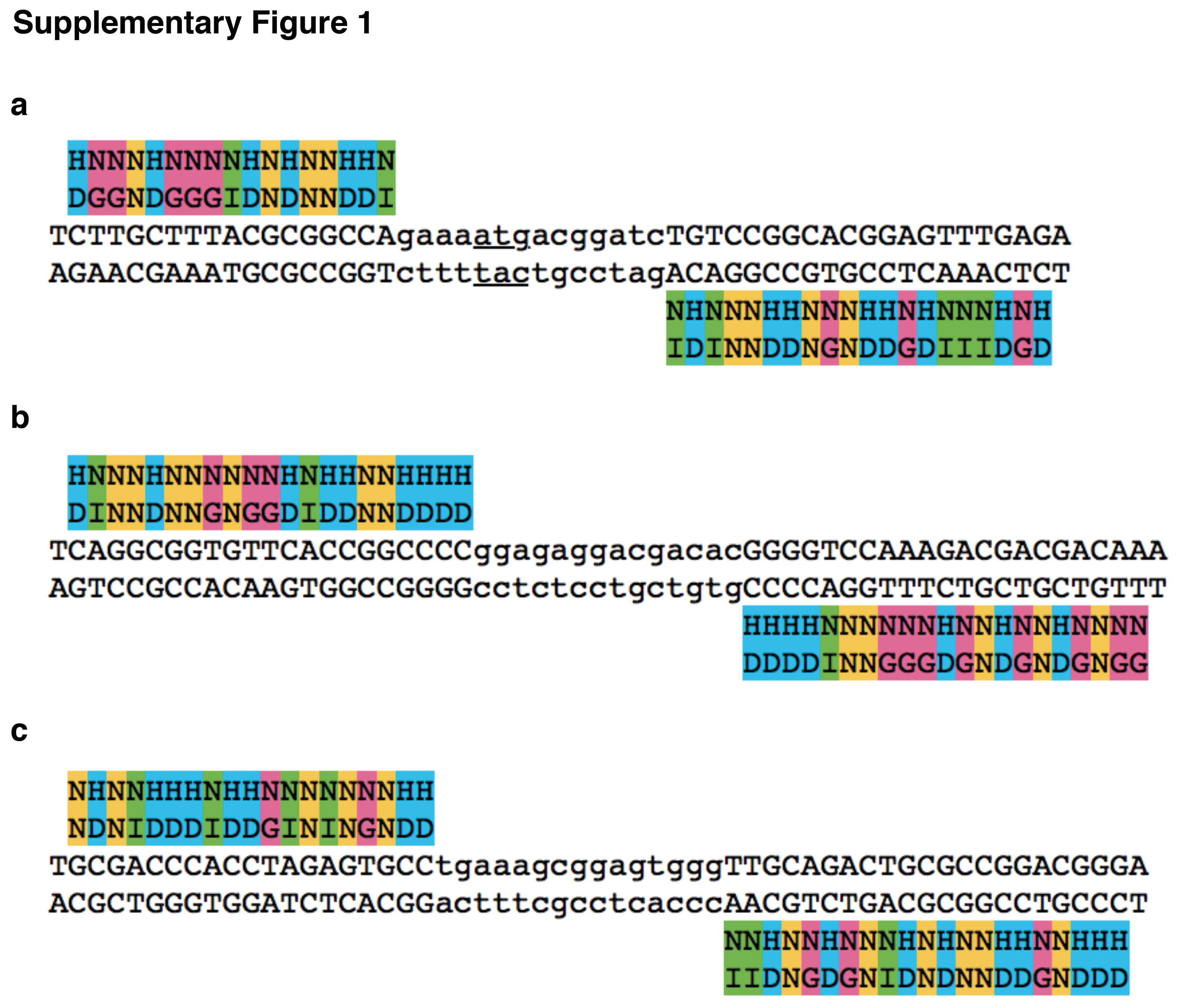

Supplement: Supplementary file 1 — Figure S1. TALEN pairs. a Alignment between TALEN P1 and dmrt2a sequence. b Alignment between TALEN P2 and dmrt2a sequence. c Alignment between TALEN P3 and dmrt2b sequence. The start codon is underlined, and the spacer region is in lowercase. Each colour represents a different Repeat Variable Diresidue (RVD). (TIF 3783 kb) [file 12861_2018_173_MOESM1_ESM.tif]

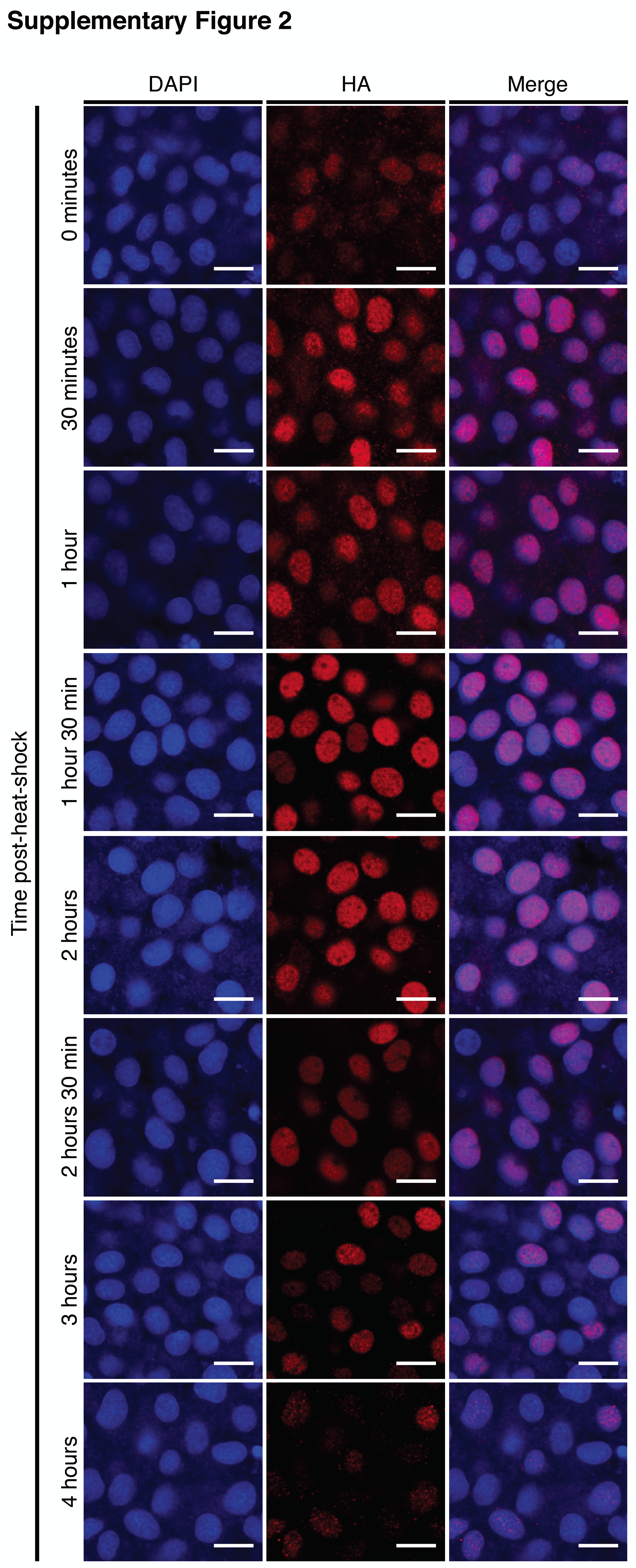

Supplement: Supplementary file 2 — Figure S2. HA-Dmrt2a dynamics after heat-shock. Immunostaining showing the expression of the HA-Dmrt2a fusion protein at different time-points post-heat-shock. The HA-Dmrt2a protein is depicted in red, and the nucleus is depicted in blue. Min: minutes. Scale bar: 10 μm. (TIF 16094 kb) [file 12861_2018_173_MOESM2_ESM.tif]

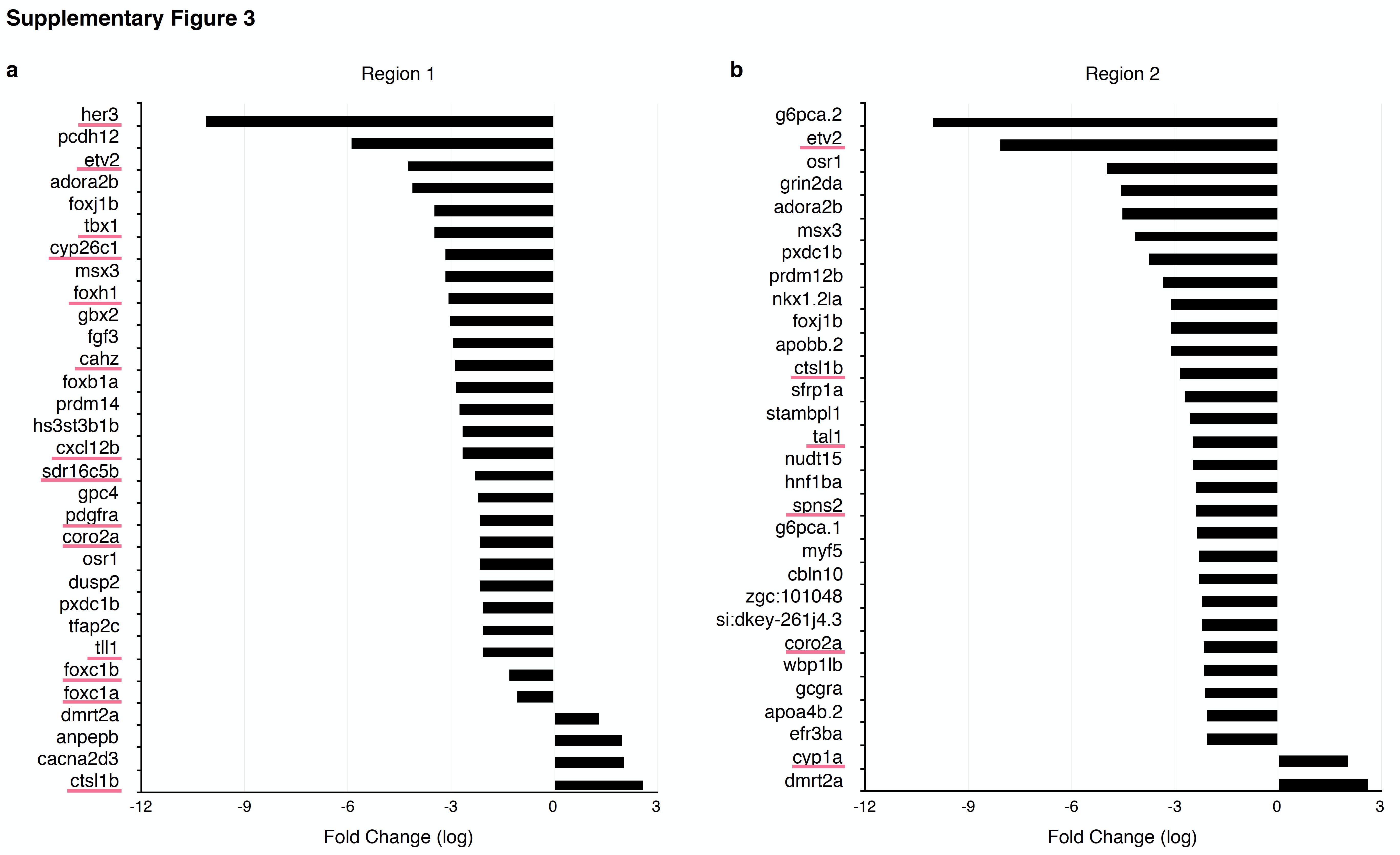

Supplement: Supplementary file 3 — Figure S3. Microarray data. a, b List of all the genes obtained in the microarray with FC > 2 or FC < − 2, P < 0.05 and FDR < 0.05, together with dmrt2a, foxc1a and foxc1b in Region 1 (FC = 1.3, P = 0.000416, FDR = 0.0677; FC = − 1.04, P = 0.219, FDR = 0.636; FC = − 1.3, P = 0.00134, FDR = 0.103, respectively). (a) Region 1 and (b) Region 2. Genes related to cardiac and vascular development are underlined. (TIF 679 kb) [file 12861_2018_173_MOESM3_ESM.tif]

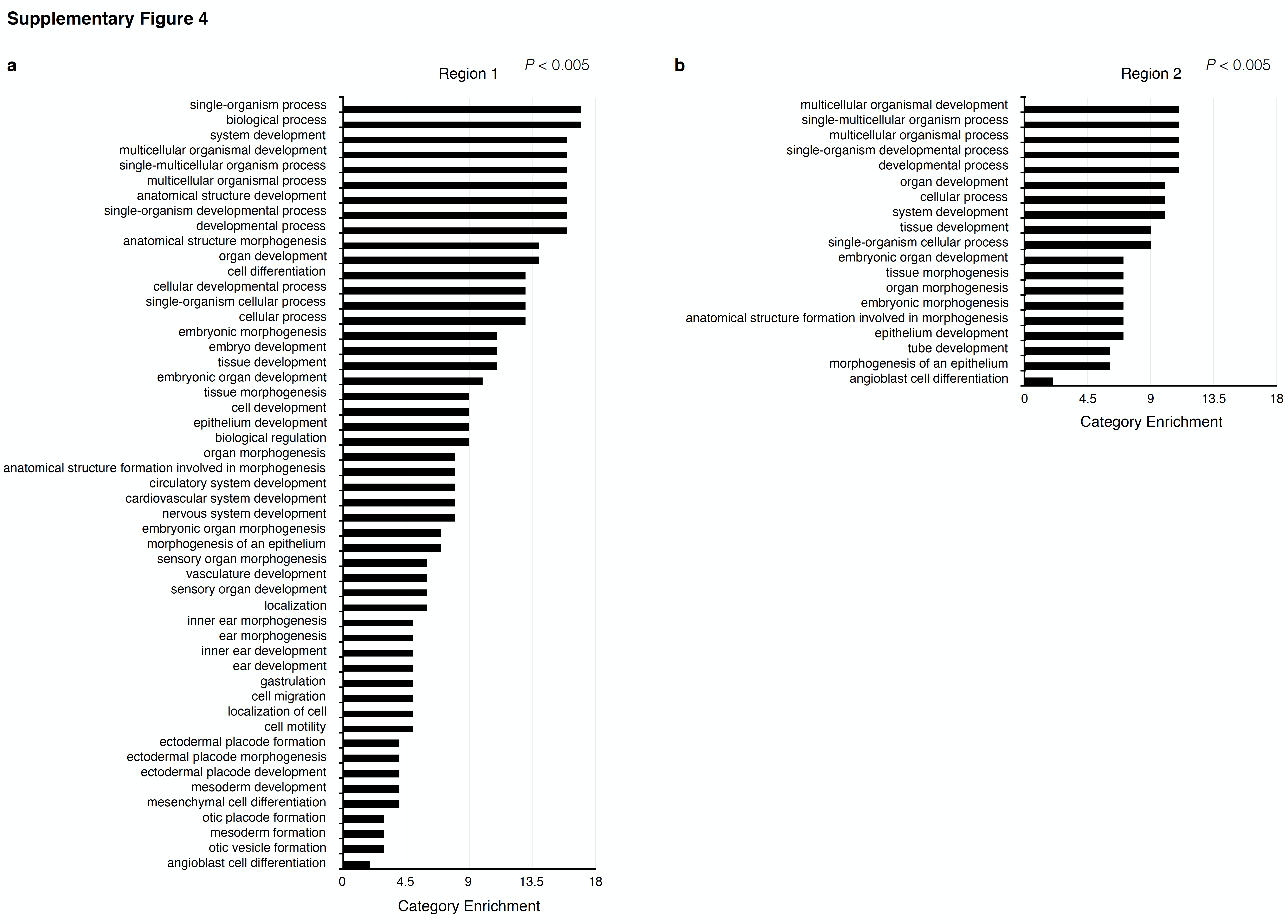

Supplement: Supplementary file 4 — Figure S4. Gene Ontology (GO) analysis of the microarray data. a, b Most significant GO terms associated with the microarray data with FC > 2 or FC < − 2, P < 0.05 and FDR < 0.05. (a) Region 1 and (b) Region 2. (TIF 1345 kb) [file 12861_2018_173_MOESM4_ESM.tif]

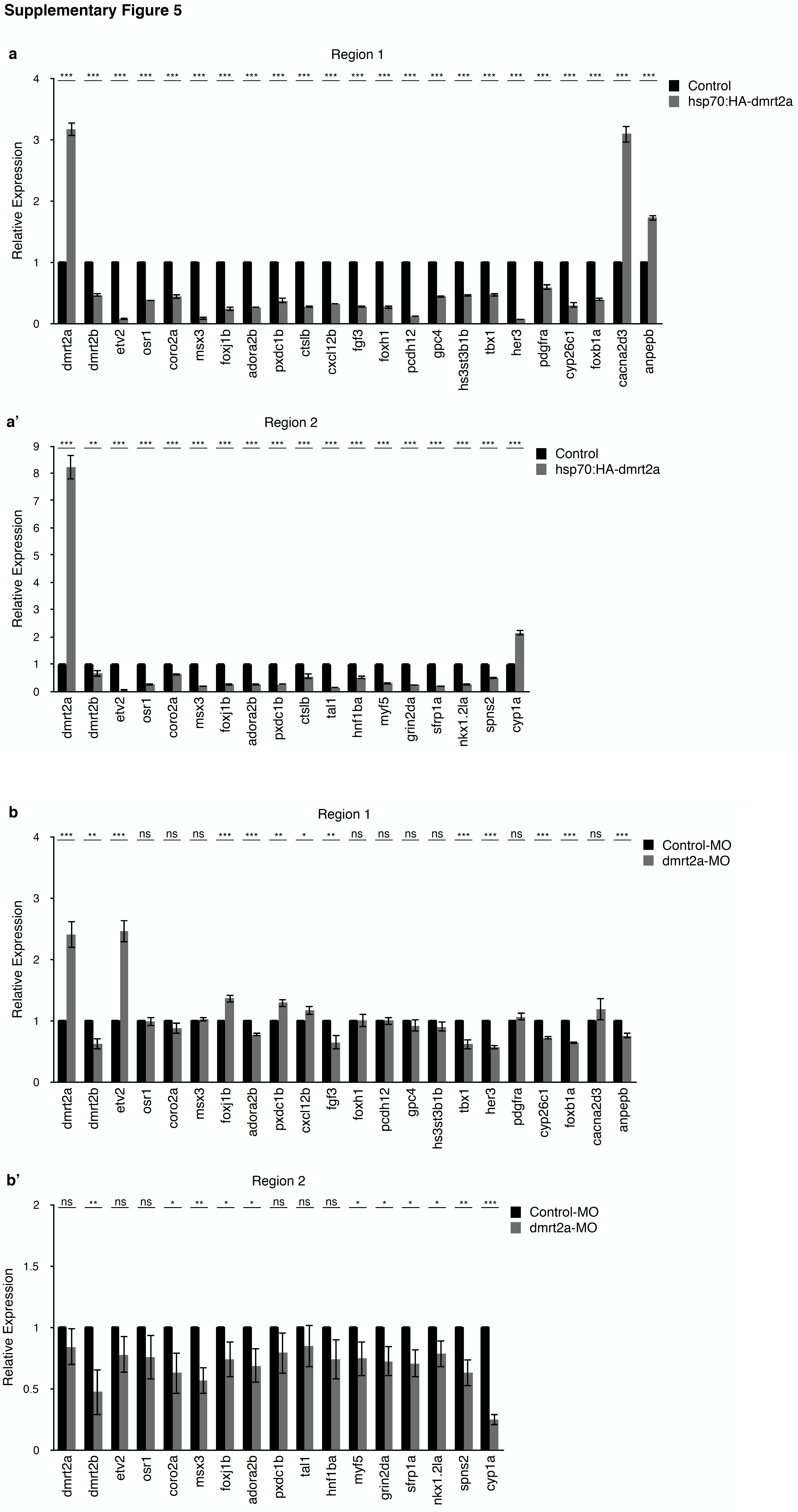

Supplement: Supplementary file 5 — Figure S5. qPCR validation of the microarray data in gain and loss-of-function experiments. a, a’ qPCR validation of selected genes from the microarray in a gain-of-function approach. (a) Region 1 and (a’) Region 2. b, b’ qPCR validation of selected genes from the microarray in a loss-of-function approach using dmrt2a-MO. (b) Region 1 and (b’) Region 2. *P < 0.05, **P < 0.01, ***P < 0.001, ns: not significant. P-values were generated using a two-tailed t-test. (TIF 1349 kb) [file 12861_2018_173_MOESM5_ESM.tif]

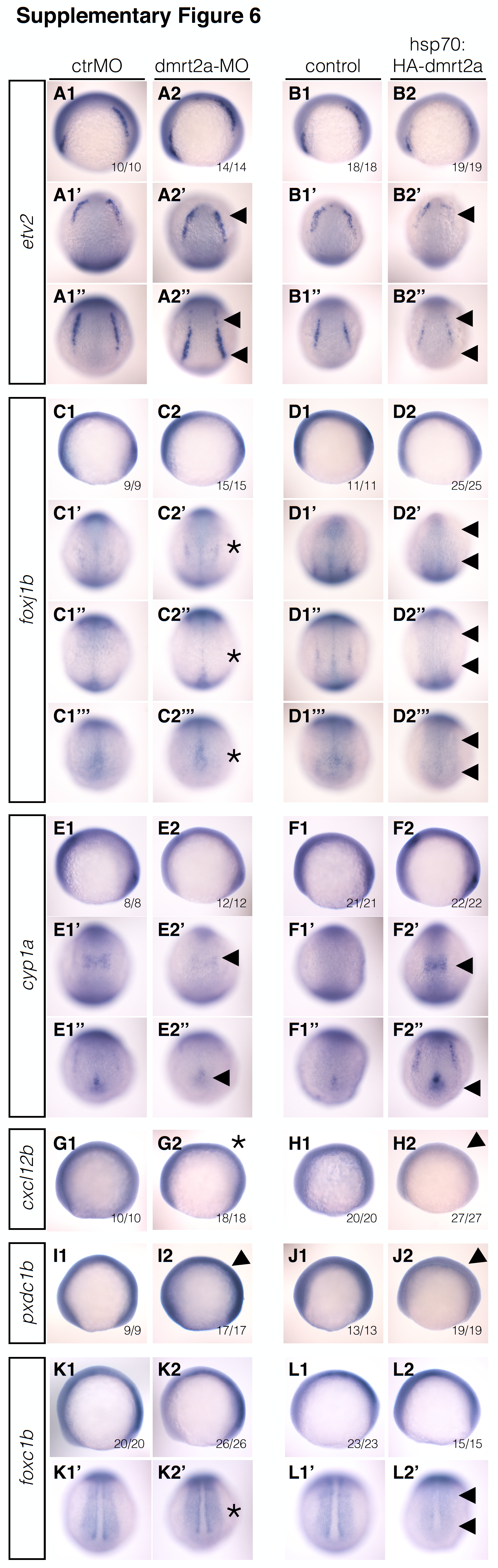

Supplement: Supplementary file 6 — Figure S6. No misexpression was found in the validated genes after dmrt2a gain and loss-of-function experiments. A1-L2’ In situ hybridisation results depicting the six genes validated in this work. A1-B2” etv2 expression pattern after injecting ctrMO (A1-A1”), dmrt2a-MO (A2-A2”), and after heat-shock in control (B1-B1”) and Tg(hsp70:HA-dmrt2a) (B2-B2”). C1-D2”’ foxj1b expression pattern after injecting ctrMO (C1-C1”’), dmrt2a-MO (C2-C2”’), and after heat-shock in control (D1-D1”’) and Tg(hsp70:HA-dmrt2a) (D2-D2”’). E1-F2” cyp1a expression pattern after injecting ctrMO (E1-E1”), dmrt2a-MO (E2-E2”), and after heat-shock in control (F1-F1”) and Tg(hsp70:HA-dmrt2a) (F2-F2”). G1-H2 cxcl12b expression pattern after injecting ctrMO (G1), dmrt2a-MO (G2), and after heat-shock in control (H1) and Tg(hsp70:HA-dmrt2a) (H2). I1-J2 pxdc1b expression pattern after injecting ctrMO (I1), dmrt2a-MO (I2), and after heat-shock in control (J1) and Tg(hsp70:HA-dmrt2a) (J2). K1-L2’ foxc1b expression pattern after injecting ctrMO (K1, K1’), dmrt2a-MO (K2, K2’), and after heat-shock in control (L1, L1’) and Tg(hsp70:HA-dmrt2a) (L2, L2’). After dmrt2a overexpression using Tg(hsp70:HA-dmrt2a) we did not observe misexpression of the six validated genes. Upon comparison with dmrt2a-MO injected embryos, we observed changes in the expression levels of some genes (arrowheads), according to qPCR data. As depicted with asterisks, after dmrt2a-MO injection we observed only very subtle changes in the expression pattern of some genes, corresponding to the less affected genes, as quantified by qPCR. All embryos were collected between 3 and 4-somite stage (loss-of-function experiments) and between 2 h and 2 h 30 min after heat-shock (gain-of-function experiments). (A1-B2, C1-D2, E1-F2, G1-H2, I1-J2, K1-L2) Lateral view, anterior to the left. (A1’-B2’, C1’-D2’, E1’-F2’) Dorsal-anterior view, anterior to the top. (A1”-B2”, C1”’-D2”’, E1”-F2”, K1’-L2’) Dorsal-posterior view, anterior to the top. (C1”-D2”) D [file 12861_2018_173_MOESM6_ESM.tif]

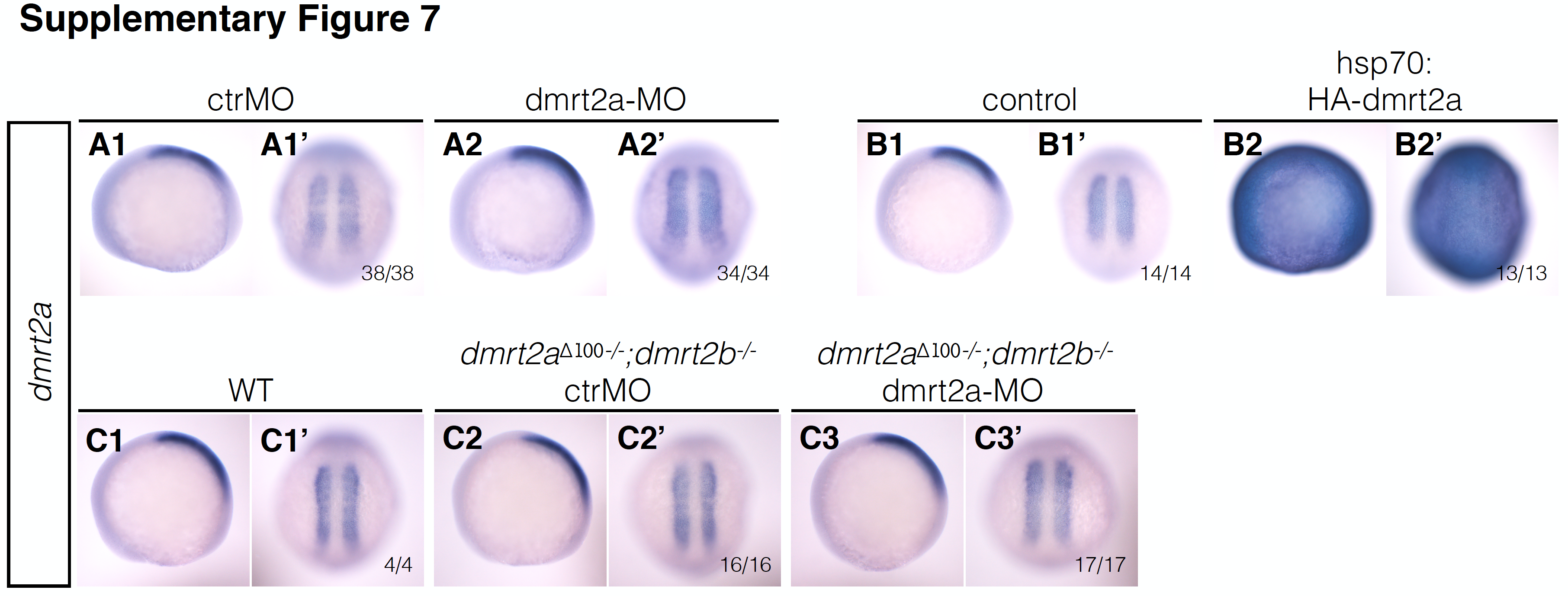

Supplement: Supplementary file 7 — Figure S7. dmrt2a expression pattern using in situ hybridisation after gain and loss-of-function experiments. A1-B2’ dmrt2a expression pattern after injecting ctrMO (A1, A1’), dmrt2a-MO (A2, A2’), and after heat-shock in control (B1, B1’) and Tg(hsp70:HA-dmrt2a) (B2, B2’). C1-C3’ dmrt2a expression pattern in wildtype embryos (C1, C1’), in dmrt2aΔ100−/−;dmrt2b−/− embryos injected with ctrMO (C2, C2’) and dmrt2a-MO (C3, C3’). (A1-C3) Lateral view, anterior to the left. (A1’-C3’) Dorsal view, anterior to the top. All embryos were collected between 3 and 4-somite stage (loss-of-function experiments) and between 2 h and 2 h 30 min after heat-shock (gain-of-function experiments). ctrMO: control morpholino, WT: wildtype. (TIF 3087 kb) [file 12861_2018_173_MOESM7_ESM.tif]

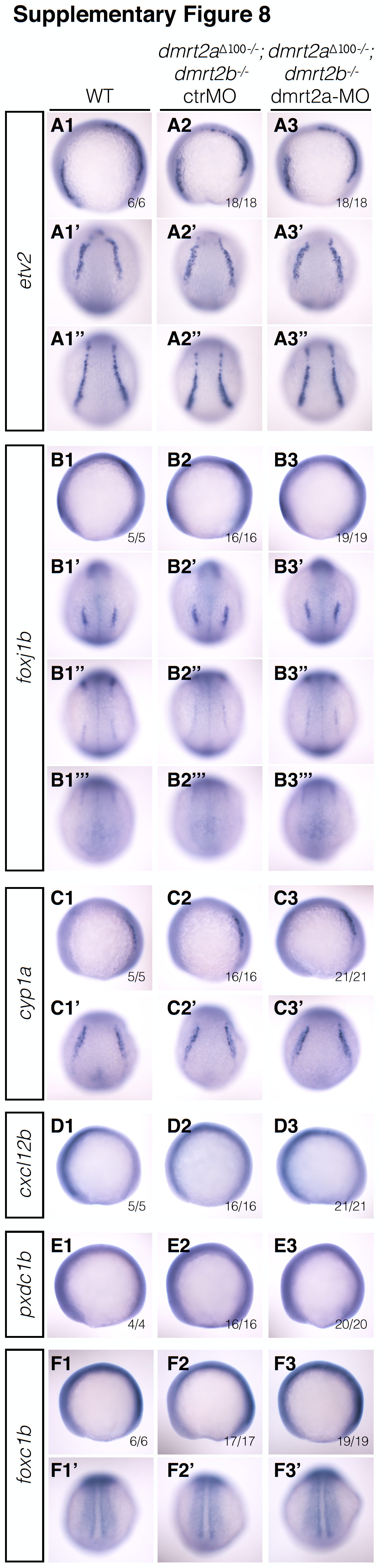

Supplement: Supplementary file 8 — Figure S8. dmrt2a-MO is specific for the validated genes. A1-F3’ In situ hybridisation results depicting the six genes validated in this work. A1-A3” etv2 expression pattern in wildtype embryos (A1-A1”), in dmrt2aΔ100−/−;dmrt2b−/− embryos injected with ctrMO (A2-A2”) and dmrt2a-MO (A3-A3”). B1-B3”’ foxj1b expression pattern in wildtype embryos (B1-B1”’), in dmrt2aΔ100−/−;dmrt2b−/− embryos injected with ctrMO (B2-B2”’) and dmrt2a-MO (B3-B3”’). C1-C3’ cyp1a expression pattern in wildtype embryos (C1, C1’), in dmrt2aΔ100−/−;dmrt2b−/− embryos injected with ctrMO (C2, C2’) and dmrt2a-MO (C3, C3’). D1-D3 cxcl12b expression pattern in wildtype embryos (D1), in dmrt2aΔ100−/−;dmrt2b−/− embryos injected with ctrMO (D2) and dmrt2a-MO (D3). E1-E3 pxdc1b expression pattern in wildtype embryos (E1), in dmrt2aΔ100−/−;dmrt2b−/− embryos injected with ctrMO (E2) and dmrt2a-MO (E3). F1-F3’ foxc1b expression pattern in wildtype embryos (F1, F1’), in dmrt2aΔ100−/−;dmrt2b−/− embryos injected with ctrMO (F2, F2’) and dmrt2a-MO (F3, F3’). We did not observe obvious differences between the three different conditions evaluated. All embryos were collected between 3 and 4-somite stage. (A1-A3, B1-B3, C1-C3, D1-D3, E1-E3, F1-F3) Lateral view, anterior to the left. (A1’-A3’, B1’-B3’) Dorsal-anterior view, anterior to the top. (A1”-A3”, B1”’-B3”’, C1’-C3’, F1’-F3’) Dorsal-posterior view, anterior to the top. (B1”-B3”) Dorsal-medial view, anterior to the top. ctrMO: control morpholino, WT: wildtype. (TIF 8561 kb) [file 12861_2018_173_MOESM8_ESM.tif]
