# Supplementary material for: Identification of Dmrt2a downstream genes during zebrafish early development using a timely controlled approach
Source: BMC Dev Biol. 2018 Jun 19;18:14. doi: 10.1186/s12861-018-0173-5 (PMC6006574; doi:10.1186/s12861-018-0173-5)
Supplement: Supplementary file 9 — Table S1. Primers used in quantitative RT-PCR. (DOC 97 kb) [file 12861_2018_173_MOESM9_ESM.doc]

**Supplementary Table 1:** Primers used in quantitative RT-PCR.

| **Gene** | **Forward sequence (5’-3’)** | **Reverse sequence (5’-3’)** |
| --- | --- | --- |
| Microdissection Experiments | | |
| *adora2b* | GGT CAC AGG TAA AAG GGC GA | TCT TTA CGG TTC CAG CCC AC |
| *anpepb* | AAA TCG CTC TGC CGG ACT TC | GGG CTG TCT CTC TGT ACG TG |
| *cacna2d3* | ACC TAT CCG TCA ACC TCA GC | ATG AGG GTT GGG TCT CTC CT |
| *coro2a* | AAC TCT GTG CCG CTA ATC CA | AGC ACC AGG AAA GAT CCA CC |
| *ctslb* | TGG AAT TAC AGT CAG TAA GGT TTG C | TCT CCT CCC GAC CTC TAC G |
| *cxcl12b* | AGC TGC CCT TTC CAA GTC ATT | CTT TGG GTT GAT GCA GAC CTC |
| *cyp1a* | AAC CAG TGG CAA GTC AAC CA | TTC AGT TCA GTA CCG TCC GC |
| *cyp26c1* | GTC TTA CCG ACA GTG CTG CT | GAT GCT CCA CCT GAA CTC CC |
| *dmrt2a-RT** | GAC AGC AAG CGA CAG AGG ACA A | CGG ATG TGT CGC TGA TAG ATA TT |
| *dmrt2b-RT* | GTG ATG GCA GCT CAA GTC GC | GGT AAG CTG TGC GTC TGA GAA |
| *elfa*** [1] | ACG CCC TCC TGG CTT TCA CCC | TGG GAC GAA GGC AAC ACT GGC |
| *etv2* | GGT TCC TGC TGG TTT CGA CTT | AAA AAG GAC AGG TCT TCA CCA C |
| *fgf3* | AAA ACA CGG AGC ACG GAC AA | CTA AGC TTG CGC ACC ATC TC |
| *foxb1a* | GGA AGG GTA GTT TCT GGG CG | GGA CAT CTG AGG GAG GTG TG |
| *foxC1a* | CGC TAT TCC GTC TCC AGT CC | CGC GTG AGA GTA CAT GGT CA |
| *foxC1b* | ACC AAG GCT TTT ACC GGA CC | AGA GTA CAT GCT CAT CGG CG |
| *foxH1* | TAG GAG AAG GAG CAC AAC GC | GCC TTG ATT CGT CGC TTT GG |
| *foxj1b* | CCA ATC CAT ACG CGC TTC AG | CTC CTC AGC CGG GTT TGT TT |
| *gpc4* | TGT GGT TAT GAG CAG CGG TT | CCG GCA TCA TCG GAG GTA AA |
| *grin2da* | TGG AAA CCC GCG ATT ACC TC | CAT ACC TGC CAC CAC CTC TC |
| *her3* | CGT CGG ACA CAG ACA GAG AT | CGC TCT CCA CGG TAC TGA AAT |
| *hnf1a* | GCT TCT GAG CGC CTT GTT GG | ATC CGT ATC GCT GAG TTT GGA |
| *hs3st3b1b* | GTA CTC ATG CGT GGG CTA CT | GGA AAT CCG TCT CCT TCG CT |
| *msx3* | AAC CTC GTA CAC TTC TCC GC | GAG CAA CTG CGA GGT GGT AA |
| *myf5* | GCA GTG TTT GTC CAG CAT CG | GGG AAT CAC TTC CGG TTG GA |
| *nkx1.2la* | GAA TCG CGC AAT GGG AAA CA | CTT CTG TGC GAG GAT GAG CA |
| *osr1* | CTC ACC CTC GTT AGG TTG CC | TCC ACG GCT GGG TTT TCT TT |
| *p53* | GGA GAC AAG CGA CTA TCC CG | CAC CAT TTG AAC GGG GCA AG |
| *pcdh12* | GCA GAT TAT GGG GCA AAC GG | CTG AAA GGG CCA GAA CCA GT |
| *pdgfra* | CGC CTA CGA CAG CAA GAG AG | ACT CGC AAA TCA GAC CCT CC |
| *pxdc1b* | CAG CCC AGC ACT CGT ATG AT | CGC TCA GGT CTT GAT CCA CAT |
| *sfrp1a* | CAA CGG ACA CCC TCC AGT TT | CGA TCC ATG TTC TCC CGC TT |
| *spns2* | CAT CTG CAG TTT CAT GGT GGC | TTC TGT TGA AGC GAT CCC CC |
| *tal1* | CCA ACA CAC CCT CCT GAC AA | GTC CCG CAC TAG AGT TGC AT |
| *tbx1* | TCC CAT TTT TGC GAT GTT GC | AAT AGT TGT AGG CGT GCT GGG |
| Evaluation of *dmrt2a* and *dmrt2b* mutant transcripts at 24 hpf | | |
| *dmrt2a-mut* | GCG ACA GAG GAC AAA AAG GGA | GGG CGG ATG TGT CGC TGA TA |
| *dmrt2b-mut* | CAG CAG GCC ACA GAG GGT AA | GTA GCG CTG GTA AGC TGT GC |

* - This primer pair was also used in the evaluation of *dmrt2a* transcripts throughout development.

** - This primer pair was used as housekeeping for all RT-PCR experiments.

**References**

1. Azevedo, A.S., et al., *The regenerative capacity of the zebrafish caudal fin is not affected by repeated amputations.* PLoS One, 2011. **6**(7): p. e22820.
